# Correcting Apparent Priming Bias Unveils Fertilizer Nitrogen‐Risk Archetypes of Surplus and Depletion Across Asian Rice Systems

**DOI:** 10.1002/advs.76227

**Published:** 2026-06-22

**Authors:** Xiuyun Liu, Siyuan Cai, Longlong Xia, Jagdish K. Ladha, Xiaoyuan Yan, Xu Zhao

**Affiliations:** ^1^ State Key Laboratory of Soil and Sustainable Agriculture Changshu National Agro‐Ecosystem Observation and Research Station Institute of Soil Science Chinese Academy of Sciences Nanjing China; ^2^ University of Chinese Academy of Sciences Beijing China; ^3^ University of Chinese Academy of Sciences Nanjing China; ^4^ Department of Plant Sciences University of California Davis California USA

**Keywords:** net residue, nitrogen loss, nitrogen management, priming effect, sustainable agriculture

## Abstract

Accurate assessment of fertilizer nitrogen (N) fate is essential for optimizing rice production, yet regional‐scale estimates remain limited. This progress has been fundamentally constrained by the prohibitive cost of isotopic tracing, which has not only limited large‐scale deployment but also prevented the correction of apparent priming effect (APE)‐induced bias, resulting in a systematic misestimation of soil N retention. To overcome this limitation, a continental‐scale framework is developed to quantify fertilizer N fate across Asian rice systems. Ensemble modeling produces the first high‐resolution (5 arcmin) maps of Net Residue and Loss, identifying Eastern and Central China, along with Northern India, as critical Loss hotspots. Accounting for APE reveals that positive priming suppresses the Net Residue of applied N to below 7% (−1%–15%), while 48% (43%–53%) of applied N is lost to the environment, leading to annual environmental costs of US$98.53 (83.25–108.27) billion from reactive‐N emissions. Crucially, three N‐risk archetypes that together encompass 42% of global rice fields emerge: 37% high‐loss/high‐net‐residue, 2% high‐loss‐soil‐depleting, and 3% low‐loss‐soil‐mining. Overall, this framework converges high‐resolution ensemble mapping, apparent priming bias correction, and policy‐oriented N‐risk archetypes to transform N governance from retrospective accounting into a spatially targeted, forward‐looking strategy reconciling food security with environmental and economic sustainability.

## Graphical abstract

Correcting the apparent priming effect resolves systematic biases in Asian rice fertilizer nitrogen accounting. Net soil retention drops below 7%, while 48% of fertilizer escapes, inflicting US$98.53 billion in annual reactive‐nitrogen damages. High‐resolution mapping uncovers N‐risk archetypes across 42% of the rice area, delivering a spatially targeted blueprint to reconcile food security with global sustainability.

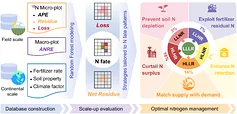

## 1 Introduction

Rice is a staple crop that underpins food security for more than half the world's population [1], yet its production depends heavily on synthetic nitrogen (N) fertilizer, consuming approximately 15% of global N fertilizer inputs [2]. Rice systems exhibit relatively low N use efficiency (NUE, 39%–45%) and face a disproportionately high risk of long‐term N losses to the environment, as indicated by national‐scale N budget analyses [3, 4]. This inefficiency translates to annual environmental costs through PM_2.5_ formation from ammonia (NH_3_) volatilization, groundwater nitrate contamination, and nitrous oxide (N_2_O) emissions [5]. Consequently, regional‐scale assessment of fertilizer N fate has emerged as a grand challenge in agricultural sustainability. The absence of spatially explicit, process‐constrained information on where fertilizer N is taken up, retained, or lost prevents governments from identifying pollution hotspots, prioritizing mitigation investments, and designing locally adapted nutrient policies. This critical knowledge gap directly hinders progress toward multiple international commitments, including Sustainable Development Goal (SDG) 2 on food security, SDG 3 on health, SDG 6 on water quality, and SDG 13 on climate action [6]. It also impedes the Kunming–Montreal Global Biodiversity Framework's target of at least halving excess nutrient losses by 2030 to reduce damage to biodiversity and ecosystem functions and services [7], as well as global efforts to curb N waste and N_2_O emissions [8]. Given that 90% of global rice production originates from Asia [9], there is an urgent need to develop sustainable N management strategies that secure yields while mitigating N pollution‐driven environmental and health risks in rice systems.

**FIGURE 1:**
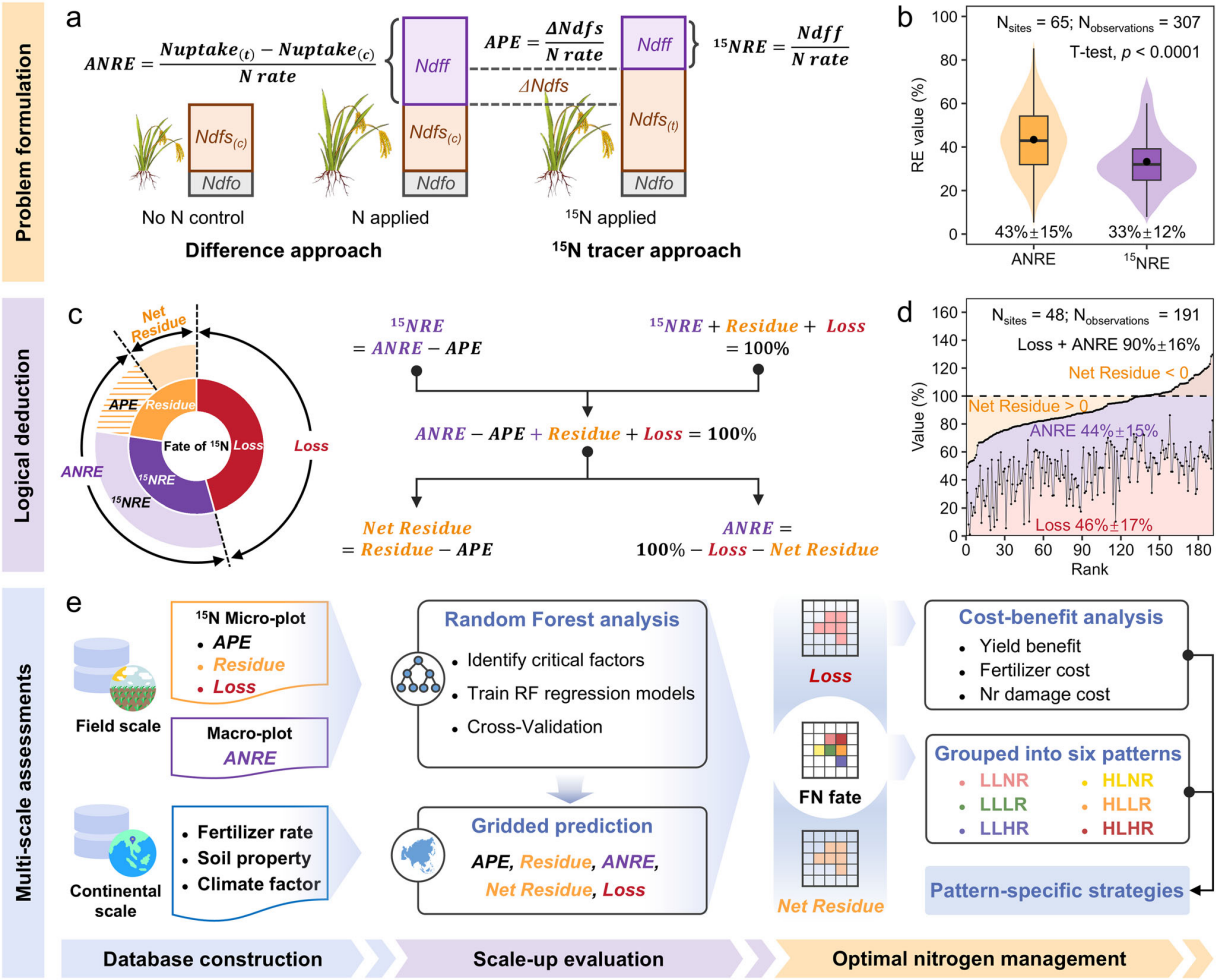
Framework for decoding fertilizer nitrogen (N) fate by leveraging the priming effect. (a) Discrepancy between apparent nitrogen recovery efficiency (ANRE) and ^15^N recovery efficiency (^15^NRE) due to changes in harvested N derived from soil (ΔNdfs), defined as apparent priming effect (APE). (b) Comparison of ANRE and ^15^NRE based on paired observations in the Meta database. (c) Quantitative relationships among ANRE, proportion of fertilizer N net residual (Net Residue; fertilizer N Residue − APE), and proportion of N loss (Loss). (d) Site‐specific experimental observations (n = 191 from 48 sites) extracted from peer‐reviewed publications that met screening criteria, arranged in descending order of the sum of ANRE and Loss. The lower‐ and upper‐fold lines show Loss and the sum of ANRE and Loss, respectively (both as % of applied N). ANRE + Loss < 100% denotes positive Net Residue (a net addition to the soil N pool by fertilizer N), whereas ANRE + Loss > 100% denotes negative Net Residue. (e) Novel framework for categorizing fertilizer N management status into six types by orthogonal thresholds: Net Residue (negative/low/high) and Loss (≤ or > 75th percentile). Categories: LLNR (Low‐Loss‐negative‐Net Residue), LLLR (Low‐Loss‐low‐Net Residue), LLHR (Low‐Loss‐high‐Net Residue), HLNR (High‐Loss‐negative‐Net Residue), HLLR (High‐Loss‐low‐Net Residue), HLHR (High‐Loss‐high‐Net Residue).

Increasing NUE in crop production is a key priority in efforts to enhance sustainable N management [10]. The primary approaches for quantifying NUE in field trials include N difference (apparent N recovery efficiency, ANRE) and ^15^N tracer (^15^N recovery efficiency, ^15^NRE) [11]. ANRE in cereal crop systems is typically significantly higher than the corresponding ^15^NRE [12, 13]. The divergence between ANRE and ^15^NRE reflects fertilizer‐induced alterations to indigenous soil N supply (Figure 1a). For Asian rice fields, we observed that an ANRE of 43% ± 15% was significantly larger than the ^15^NRE of 33% ± 12% (Figure 1b), indicating that synthetic N fertilizer promoted the uptake of native soil‐derived N in aboveground sections of rice. The phenomenon whereby plants supplied with fertilizer N exhibit enhanced N uptake from soil in comparison to those not receiving N fertilization is referred to as the apparent priming effect (APE) or added N interaction (ANI) [14], a term first introduced by Bingeman et al. (1953) [15]. It arises through three partially overlapping mechanisms. Fertilizer N accelerates root proliferation, enlarging the volume of soil explored, thereby enhancing access to native N [16]; it simultaneously drives cation exchange, in which fertilizer‐derived ^15^NH_4_^+^ displaces sorbed or complexed native NH_4_^+^ and disrupts Ca_2_^+^‐mediated bridging in organomineral associations, accelerating organic N decomposition [17]. Moreover, by increasing rhizosphere carbon inputs that fuel microbial metabolism—and by directly stimulating microbial activity and extracellular enzyme production—fertilizer N further accelerates depolymerization and mineralization of organic N [18]. These mechanisms imply that fertilizer application affects not only the fate of the applied N but also the cycling of the native soil N pool.

Although ^15^N microplot experiments enable accurate tracing of fertilizer N fate in the soil‐crop system [19], ignoring APE leads to overestimation of soil N replenishment by fertilizer‐derived N (Figure 1c). Specifically, the replenishment effect of the ^15^N‐labeled N that appears to be retained in the soil is, in reality, partly offset by the concurrent removal of native soil N that is released through the priming effect and subsequently taken up by the crop. Most field studies and regional N budgets still ignore APE, implying that current assessments of fertilizer‐induced changes in soil N pools are systematically biased. To address this, we introduce the Net Residue—calculated as Residue minus APE—as a correction term within the ^15^N‐based mass‐balance framework. Net Residue rectifies the misestimation inherent in Residue alone and, together with ANRE and Loss, satisfies the algebraic closure of the fertilizer N fate equation: ANRE + Net Residue + Loss = 100% (Figure 1c). It should be noted that Net Residue does not capture the full impact of fertilization on the total soil N balance, because the priming effect may also accelerate the loss of native soil N—a flux not explicitly resolved in the current framework, as APE is built upon fertilizer‐derived ^15^N budgets that inherently track only the partitioning of the applied N among crop uptake, soil residue, and environmental loss. Consequently, Net Residue is interpreted here as the net retention of fertilizer‐derived N in the soil within a single growing season, rather than a direct measure of long‐term soil N stock change.

Crucially, because ANRE, Net Residue, and Loss are constrained to sum to 100% (Figure 1c), any increase in one flux must be compensated by a decrease in the others. This means that NUE is governed by a direct trade‐off between the net retention of fertilizer‐derived N in the soil (Net Residue) and the loss of fertilizer‐derived N to the environment (Loss). These two dimensions jointly determine the agronomic and environmental performance of N management. Theoretically, when APE and Residue mutually offset, the soil N pool attains a quasi‐steady state due to the absence of Net Residue, rendering ANRE determined solely by Loss. However, empirically, our compiled dataset across Asia (n = 191) revealed that the sum of ANRE and Loss commonly deviated from 100% (Figure 1d), with 72% of cases falling below 100% (positive Net Residue, indicating net fertilizer‐derived N retention) and the remaining 28% exceeding 100% (negative Net Residue, indicating a net deficit of fertilizer‐derived N in the soil). The average Net Residue was 10% ± 16%, suggesting that in‐season fertilizer N retained in the soil mostly outweighed the crop's additional uptake of soil N induced by synthetic N fertilizer across Asian rice production. Alongside Loss, Net Residue thus constitutes a second critical metric for diagnosing N management status—one that reveals whether current practices are building N stock in the soil or eroding it. However, most national nutrient balance reports still treat soil N balance and loss pathways in isolation [6, 10], often quantifying recovery efficiency while leaving the loss and residue terms poorly constrained [20]. This fragmented approach produces interventions that optimize one dimension at the expense of the other, for example, rate reductions that lower losses but may exacerbate hidden deficits in Net Residue, or practices that boost soil N retention but amplify downstream emissions. A multi‐objective optimization framework for fertilizer N management in regional rice systems that balances enhanced NUE, maintains soil fertility, and reduces environmental risks from N losses remains largely underdeveloped.

Motivated by the urgent need for regional data on synthetic fertilizer N management status to facilitate sustainable rice intensification, we established the first high‐resolution mapping framework for fertilizer N fate in Asian rice systems (Figure 1e; Figure S1). The process utilized robust historical datasets compiled from 570 monitoring observations from ^15^N tracer micro‐plot trials and 5584 observations from macro‐plot trials (N difference method) (Figures S2–S4). First, Random Forest models were constructed to quantify the relative importance of drivers of APE, Residue, ANRE, Net Residue, and Loss, and to map their spatial patterns (Figure S5). Subsequently, thresholds were imposed on Net Residue and Loss to delineate six fertilizer N management archetypes in Asian rice fields. Finally, by integrating cost‐benefit analysis of synthetic fertilizer N input, tailored optimization strategies were prescribed for distinct fertilizer N fate patterns to operationalize sustainable N management across Asian rice systems.

## 2 Results and Discussion

### 2.1 Spatial Patterns of Net Residue Arise from the Balance Between APE and Residue

**FIGURE 2:**
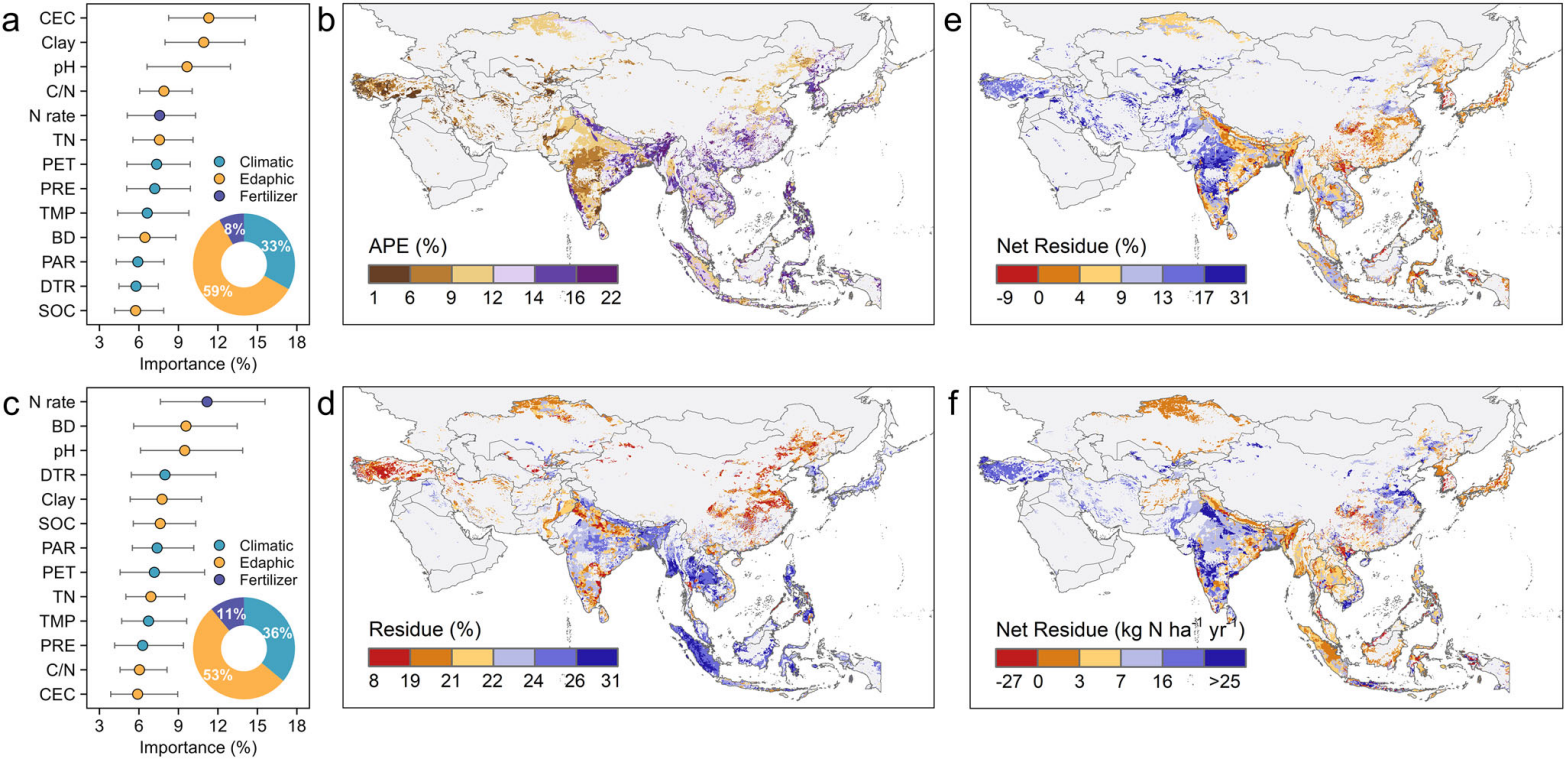
Spatial patterns and drivers of fertilizer nitrogen (N) dynamics in Asian rice systems. The relative importance of drivers for the apparent priming effect (APE) (a) and fertilizer N residue (Residue) (c) variation, as identified by Random Forest regression. Predicted spatial distributions of APE (b), Residue (d), and proportion of net residual fertilizer nitrogen (Net Residue) (e). (f) Gridded annual net residual fertilizer N per unit area. Fertilizer factors: N rate, nitrogen application rate (kg N ha^−1^). Climatic factors: TMP, daily mean temperature (°C); DTR, diurnal temperature range (°C); PAR, photosynthetically active radiation (W m^−2^); PRE, precipitation (mm month^−1^); PET, potential evapotranspiration (mm day^−1^). Edaphic factors: BD, bulk density (g cm^−3^); Clay, percent by weight clay (%); CEC, cation exchange capacity (cmol kg^−1^); pH, pH measured in a soil‐water solution (−log(H^+^)); SOC, soil organic carbon (g kg^−1^); TN, total nitrogen content (g kg^−1^); C/N, carbon‐to‐nitrogen ratio.

Positive APE induced by synthetic N fertilizer was commonly observed in Asian rice fields, ranging between 1% and 22% of applied N, dominated by soil properties across Asian rice systems (Figure 2a,b). Values clustered below 9% in the arid irrigated valleys of West Asia—such as Iran, Iraq, and Afghanistan—as well as the dry plains of Western India and the loess‐derived uplands of Northwestern China (Figure 2b; Figure S6). The soils are dominated by large cation exchange capacity (CEC; 25–30 cmol kg^−1^) and clay fractions that strongly sorb NH_4_^+^ and suppress the acid hydrolysis and enzymatic depolymerization that liberate native organic N (Figure S7) [21, 22]. The pattern reversed in the humid lowlands of Southeast Asia, where Vietnam, Thailand, Cambodia, and the riparian lowlands of Indonesia and the Philippines showed APE over 14 % (Figure 2b). There, low‐CEC Acrisols and Gleysols tended to hold less NH_4_^+^ on exchange sites, increasing fertilizer N availability in solution, while vigorous rhizosphere exudation and rapid microbial turnover provided labile carbon that can intensify native N cycling and, in some settings, biological N fixation [23, 24]. The results advance two fronts. First, they provide empirical evidence that the magnitude of fertilizer‐induced priming is predictable from measurable soil attributes, which can be embedded in next‐generation Earth‐system and crop models to minimize uncertainty in regional N‐fate forecasts. Second, APE offers a missing link between short‐term agronomic gains and long‐term soil organic carbon (SOC) trajectories. High‐APE regions may achieve impressive ANRE today, yet they risk accelerated soil N depletion and heightened N losses tomorrow—a trade‐off invisible under conventional NUE metrics. The positive APE resulted in significantly greater ANRE compared to ^15^NRE (*p* < 0.0001). The mechanistic disparities between these two NUE metrics are detailed in Text S1. The spatial pattern and environmental drivers of ANRE (Figure S8) are detailed in Text S2.

Fertilizer N Residue displayed a clear latitudinal gradient across Asian paddy systems, ranging between 8 and 31% of the applied N (Figure 2c,d). High‐Residue hotspots coincided with lower‐input (<50 kg N ha^−1^), yet clay‐rich paddies—e.g., the Ganges–Brahmaputra delta, Myanmar, eastern India, and the lower Mekong and Red River deltas—where alluvial soils (low bulk density and high CEC) are known to fix NH_4_^+^ between 2:1 clay layers, thereby buffering seasonal N surpluses [25]. Mechanistically, fertilizer NH_4_^+^ outcompetes exchangeable potassium (K^+^) for interlayer sites due to their similar ionic radii and lower hydration energy, forming a fixed NH_4_^+^ pool that curbs in‐season Losses and elevates Residue [26]. This ‘clay cache’ is most effective under mildly acidic to neutral conditions typical of central Indian Vertisols, which display high Residues yet low APE (Figure 2b,d). Fertilizer application rate emerged as the strongest predictor of Residue, accounting for one‐tenth of the total variable importance (Figure 2c). Regions with high fertilizer N inputs (>150 kg N ha^−1^)—such as West Asia and northwestern East Asia—exhibited low APE and Residue, whereas lower‐input regions in western Southeast Asia demonstrated higher APE and Residue (Figure 2b,d). Climate variables contributed the remaining one‐third of the variable importance. Residue complements APE by illuminating the storage rather than mobilization side of soil N dynamics; together they span the assimilation–mineralization continuum and advance N‐cycle modeling.

Net Residue integrates Residue and APE into a systems‐balance metric that indicates whether fertilization is building, maintaining, or depleting the soil N pool, providing actionable guidance for N stewardship across Asian rice systems. Asian rice systems exhibited high spatial variability in Net Residue, with predicted values ranging from −9% to 31% (−27–133 kg N ha^−1^ year^−1^). Despite high Residue in the rice fields of southern and eastern China, Southeast Asia, and the eastern regions of South Asia (Figure 2d), substantial APE largely offset fertilizer N replenishment to soil pools. Consequently, Net Residue fell below 9% in these regions, with scattered hotspots of negative Net Residue posing soil N depletion risks (Figure 2e). Fields characterized by negative Net Residue accounted for 5% of Asia's total rice harvested area. Such deficits imply that apparent N Residue is insufficient to offset the priming‐induced plant uptake of native soil N. Consistent with this mechanism, a long‐term Japanese paddy showed year‐on‐year decreases in both SOC and TN [27], a pattern diagnostic of the soil‐depleting archetype, although other management and environmental drivers may additionally contribute. In contrast, rice fields with high Net Residue were located mainly in western India and western Asia, attributed to high soil CEC and high photosynthetically active radiation (PAR), respectively (Figures S6 and S9). Annual Net Residue exceeded 25 kg N ha^−1^ in the eastern North China Plain, the western Indo‐Gangetic Plain, the southwestern Deccan Plateau, and the Mekong Delta, driven primarily by excessive N application. Independent evidence reinforces this diagnosis, revealing that in Western India, total soil N rose in tandem with a 0.8 t C ha^−1^ yr^−1^ gain in organic C pool over a decade [28]. A meta‐analysis of synthetic‐N additions likewise documented increases in SOC of 3%–16% and organic N of 8%–15%, with the largest gains in tropical environments [29].

### 2.2 Losses Offset a Quarter of Asian Rice Production Gains

**FIGURE 3:**
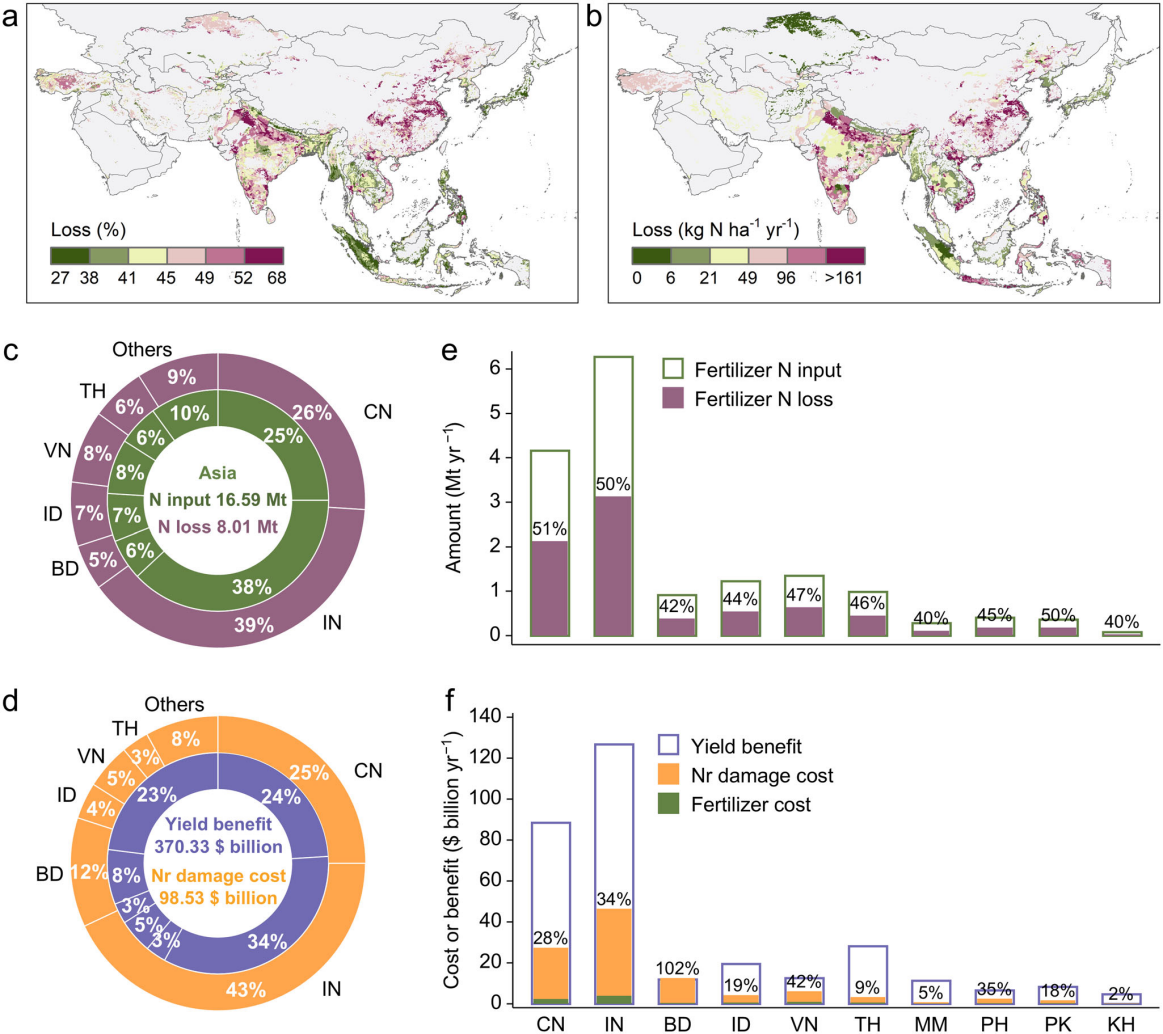
Spatial patterns and cost‐benefit analysis of synthetic fertilizer nitrogen (N) losses in Asian rice systems. (a) Predicted in‐season proportions of fertilizer N loss using Random Forest regression. (b) Gridded annual fertilizer N losses per unit area. (c) National distribution of annual fertilizer N input (dark green) and losses (dark purple) with Asian totals (million tons, Mt). (d) Country‐specific distribution of annual yield benefit (purple) and reactive N (Nr) cost (orange) with Asian totals. (e) Fertilizer N input and losses for the top 10 Asian rice‐producing countries. Percentages show the proportions of N loss to the environment. (f) Cost‐benefit analysis of yield benefit, Nr damage cost, and N fertilizer cost in 2020. Percentages represent the proportions of Nr damage cost to the yield benefit. The top 10 rice‐producing Asian countries ranked by descending 2020 output: China (CN), India (IN), Bangladesh (BD), Indonesia (ID), Vietnam (VN), Thailand (TH), Myanmar (MM), Philippines (PH), Pakistan (PK), and Cambodia (KH) (FAO 2022). Cost‐benefit data for other countries can be found in Dataset S1.

We estimated total fertilizer‐derived losses as the residual ^15^N not recovered in crop or soil pools (see Methods). Although the proportions of synthetic fertilizer N losses varied substantially across Asian rice systems, ranging from 27% to 68%, their spatial hotspots demonstrated pronounced geographic concentration (Figure 3a). Regions with elevated fertilizer N losses mainly occurred in Eastern, Central, and Northwestern China, Northern and Southern India, and Northern Vietnam (Figure 3a,b). The spatial hotspots showed strong congruence with documented critical reactive N (Nr) emission zones [30], including machine learning‐derived ammonia emissions [31], process‐based simulated N_2_O emissions, and nitrate leaching across croplands [32]. Substantial fertilizer N losses exceeding 161 kg N ha^−1^ year^−1^ were driven primarily by high N application rates (Figure 3b; Figures S6 and S10), aligning with dominant factors identified for agricultural N losses in prior DeNitrification‐DeComposition model simulations [33]. Consistent with the Law of Diminishing Returns, increasing the N application rate reduced ANRE and Residue while amplifying Loss [34, 35]. This indicated that the decline in NUE in high‐input zones was propelled by more than the in‐season Nr Losses or the physiological dilution of ^15^N recovery under surplus supply [36, 37, 38]. Equally important is a tightening or loosening of the APE–Residue balance. Where APE offset Residue, Loss, alone governed ANRE; where Residue accumulated without an equivalent priming drawdown, surplus soil buffering capacity damped marginal Loss. This explains the central Indian “efficient–retentive” rice field hotspots (Figure S8b; Figures 2e and 3a), wherein enhanced soil N buffering capacity sustains high ANRE by decoupling genuine fertilizer N retention from priming‐induced native N loss. Moreover, climatic factors influenced spatial patterns of Loss, with PAR negatively associated with Loss (Figure S10b). This suggests that favorable climatic conditions, particularly elevated PAR, enhance rice biomass production and concomitant N uptake, which competitively deplete the soil mineral N pool and thereby attenuate fertilizer N Loss to the environment.

Our study advances previous research by establishing a high‐resolution map of total fertilizer N loss across Asian rice systems, enabling a comprehensive cost‐benefit analysis of fertilizer N inputs. To account for variability in damage cost valuations, we adopted a unified three‐scenario framework comprising a low‐cost scenario, a baseline (median‐cost) scenario, and a high‐cost scenario (see Text S3 for details on scenario construction). In 2020, Asian rice systems lost 8.01 Mt N yr^−1^—approximately 48% of the fertilizer N they received (Figure 3c). These losses encompassed 5.09 Mt N yr^−1^ of Nr emissions, primarily as NH_3_ volatilization (3.77 Mt N yr^−1^), N_2_O emissions (0.11 Mt N yr^−1^), nitrogen oxides (NO_X_) emissions (0.04 Mt N yr^−1^), leaching (0.67 Mt N yr^−1^), and runoff (0.50 Mt N yr^−1^) (Figure S11b). Under the baseline scenario, the total environmental damage costs of these Nr emissions reached US$98.53 billion yr^−1^, which offset 27% of the yield benefit in Asian rice production (Figure 3d). The sensitivity analysis yielded a bounded range of US$83.25–108.27 billion yr^−1^, with total environmental costs at the lower bound under the low‐cost scenario and at the upper bound under the high‐cost scenario (Figure S12a,b). The corresponding net societal benefits—defined as the difference between yield revenue and the combined costs of N fertilizer inputs and Nr‐induced environmental damage—were US$277.26 billion yr^−1^ (low‐cost), US$261.98 billion yr^−1^ (baseline), and US$252.23 billion yr^−1^ (high‐cost) (Figures S11d and S12c,d).

From a national perspective, China and India, the leading rice producers in Asia, accounted for 63% of the total fertilizer N inputs, which in turn contributed 58% of the overall yield benefits (Figure 3c,d). The highest levels of Loss occurred in India (3.13 Mt N yr^−1^), followed by China (2.12 Mt N yr^−1^), suffering Nr costs of US$42.47 billion yr^−1^ and US$25.04 billion yr^−1^, respectively (Figure 3e,f). These two countries were responsible for 68% of the Nr damage costs in Asian rice production, highlighting the urgency of Nr emission mitigation via N rate optimization [39]. In Bangladesh, despite a modest fertilizer N loss rate of merely 42%, the total Nr damage cost (US$12.14 billion) exceeded the rice production benefit (US$11.86 billion) under the baseline scenario. As the most densely populated country (1,278 persons km^−^^2^ in 2020) among Asian nations, with over 100 million inhabitants exposed to fertilizer‐derived Nr emissions [40], Bangladesh incurred annual health damage costs of US$11.85 billion—nearly entirely offsetting the economic value of rice production (Figure S11d). Conversely, in Myanmar and Cambodia, where population densities were 81 and 95 persons km^−2^ in 2020, respectively, Nr‐induced health damage costs accounted for only 2% of the yield revenue. These nations emerged as the most efficient rice producers, with net benefit ratios (94% and 98%) substantially surpassing the Asian average (71%). Significant economic trade‐offs exist in low rice‐price economies. In nations with low rice prices, where the farm‐gate rice price was less than half of the Asian average, substantial costs of Nr losses were estimated to negate over 35% of the yield revenue (Figure 3f), as observed in countries such as Vietnam and the Philippines. Consistently, NUE exhibits a U‐shaped association with GDP, providing indirect evidence that lower‐income regions are more prone to N losses under suboptimal management [41].

### 2.3 Loss‐Net Residue Guides Optimization of Rice N Management

Optimizing N management in Asian rice requires a holistic understanding of fertilizer N fate—partitioning into crop uptake, soil retention (Net Residue), and environmental losses. We propose that the ideal sustainability benchmark achieves simultaneous loss reduction and soil fertility maintenance, distinct from systems where losses dominate, soils are being mined, or surplus N is being buffered temporarily. To this end, we developed a bivariate diagnostic framework by jointly thresholding Loss (low, high; 75th percentile) and Net Residue (negative, low, high; 0 and 75th percentile), which classifies rice systems into six fertilizer N fate types (Figure S13). Abbreviations and definitions of the fertilizer N fate types are provided in Table S1.

**FIGURE 4:**
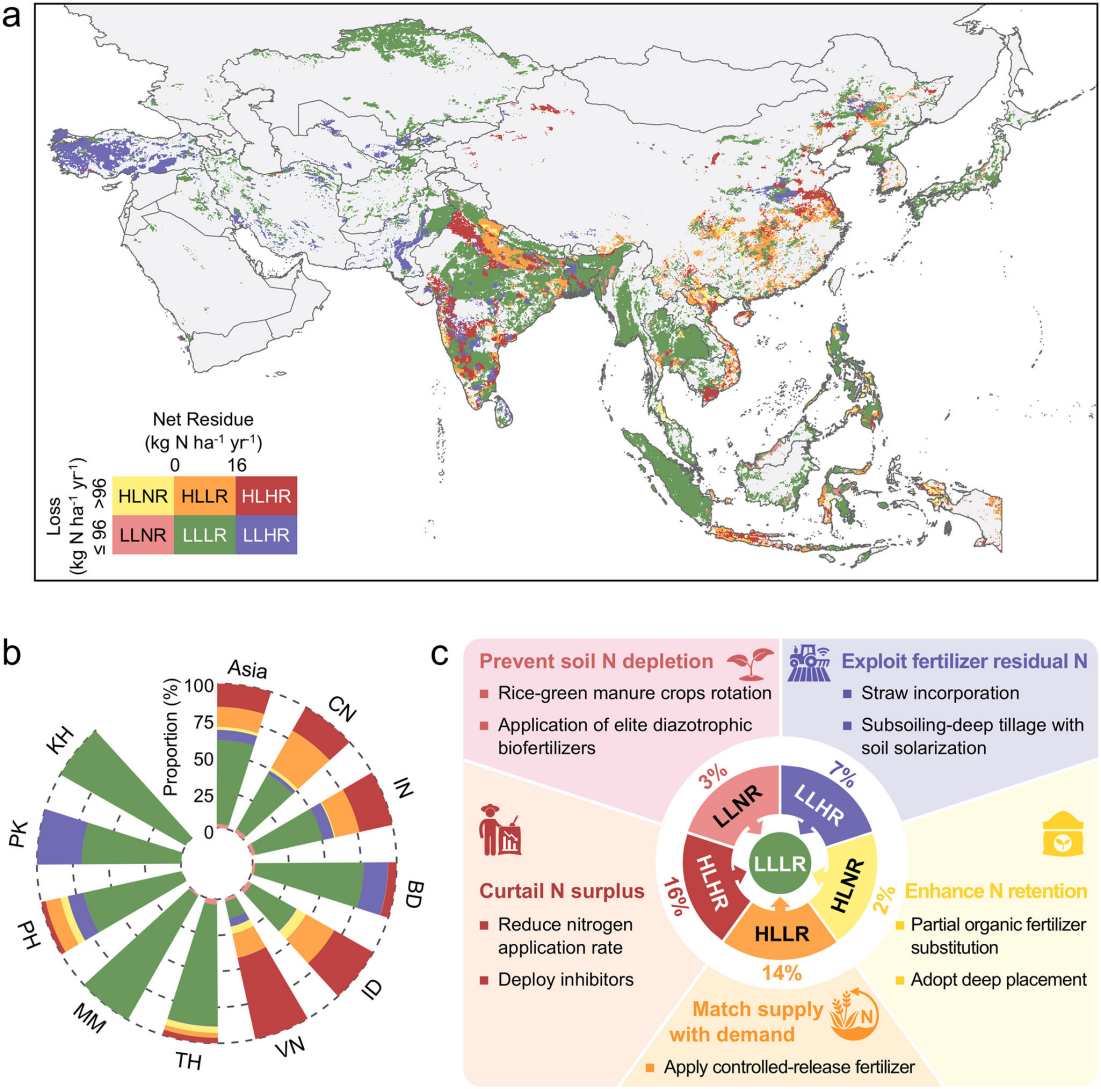
Fertilizer nitrogen (N) management status across Asian rice systems. (a) Patterns of fertilizer N management are categorized into six types: LLNR, low‐Loss‐negative‐Net Residue; LLLR, low‐Loss‐low‐Net Residue; LLHR, low‐Loss‐high‐Net Residue; HLNR, high‐Loss‐negative‐Net Residue; HLLR, high‐Loss‐low‐Net Residue; HLHR, high‐Loss‐high‐Net Residue. (b) National areal distribution of fertilizer N fate patterns for the top 10 rice‐producing Asian countries. Top 10 rice‐producing Asian countries ranked by descending 2020 output: China (CN), India (IN), Bangladesh (BD), Indonesia (ID), Vietnam (VN), Thailand (TH), Myanmar (MM), Philippines (PH), Pakistan (PK), and Cambodia (KH) (FAO 2022). (c) Asian pattern‐specific optimization strategies for N management. The percentage near the circle denotes a pattern‐specific area distribution across Asian rice systems. The upper‐quartile criterion is used to classify rice systems based on Net Residue threshold: negative, low, and high; similarly, Loss was categorized as low or high (Figure S13).

Applying the dual‐dimensional Loss‐Net Residue framework to high‐resolution (5 arcmin) maps for 2020 revealed striking spatial heterogeneity in N management status across Asia (Figure 4a). Characterized by high Loss and low efficiency, rice systems under high fertilizer N inputs were predominantly classified as high‐loss types. The HLHR (high‐Loss‐high‐Net Residue) type, representing intensive yet inefficient systems with elevated pollution risks, accounted for 16% of Asian rice area and was mainly located in Eastern and Northwestern China, Northern and Southern India, the Mekong Delta, and Southern Indonesia (Figure S14 and Text S4). HLLR (high‐Loss‐low‐Net Residue) areas, where over‐application exacerbated fertilizer N losses with little soil buffering, covered 14% of Asian rice land, prevalent in Central and Eastern China, Northeastern India, and Central Vietnam. Notably, high‐Loss‐negative‐Net Residue (HLNR) systems, facing both severe losses and a net deficit of fertilizer‐derived N in soil, implying that current management fails to sustain even the fertilizer‐derived fraction of soil N capital, comprised 2% of the area.

Conversely, low‐input systems are typically associated with high efficiency. The LLLR (low‐Loss‐low‐Net Residue) type, an ideal state of efficient production with minimal environmental impact and no net deficit of fertilizer‐derived N in soil, was the most widespread (58% of the area), prominent in Southeast Asia, central and eastern India, and Japan. Myanmar and Cambodia exemplified this type, achieving clean production with environmental costs of Nr constituting about 5% and 2% of yield benefits, respectively (Figure 3f). Scattered low‐Loss‐high‐Net Residue (LLHR) regions (7% of total area)—in Henan Basin in China, central India, and the irrigated cereal belts of Turkey, Iran, and Pakistan—exhibited considerable net retention of fertilizer‐derived N in soil. In contrast, scattered low‐Loss‐negative‐Net Residue (LLNR) areas (3% of the total area) in South Korea and Eastern India exhibited a persistent net deficit of fertilizer‐derived N in soil. Despite minimal environmental losses, these systems are actively mining native soil N through priming‐induced mineralization, indicating that low N Loss alone masks progressive soil fertility degradation and cannot sustain long‐term soil N supply.

Our dual‐dimensional analysis indicated that nearly half (42%) of Asian rice area required optimized N management, with persistent high‐loss hotspots—particularly China, India, Indonesia, and Vietnam—demanding prioritized intervention (Figure 4b). Effective strategies must be region‐specific and align with dominant fertilizer N fate patterns (Figure 4c). In HLHR‐type rice systems of developing countries such as India and Vietnam, where high input costs erode net benefits despite robust soil fertility, management should exploit this inherent retention capacity by reducing fertilizer rates (calibrated via soil N testing) and deploying nitrification inhibitors to block surplus‐driven loss pathways [42]. Where net benefit is already high, such as in Indonesia, technology‐based mitigation measures like gene editing technologies in rice breeding [43], smart fertilizers, sensor‐based variable rate application systems, AI‐driven decision support systems (DSS), and Internet of Things (IoT)‐enabled precision irrigation platforms could facilitate sustainable agriculture further. Early evidence from South Korea's Smart‐Farm 4.0 program and California's climate‐smart rice initiative shows that such digital‐ecological bundles can reconcile farm income with planetary N limits [44]. Furthermore, financial incentives must be precisely targeted [39].

Rice fields with negative Net Residue—indicating a net deficit of fertilizer‐derived N in soil—should receive replenishment credits to incentivize practices that promote the replenishment of fertilizer‐derived N in the soil (e.g., legume or green‐manure integration, partial straw return, compost/manure additions), conditional on maintaining low external Losses [45]. Conversely, areas with high Net Residue face legacy N accumulation. Blanket input subsidies that depress fertilizer prices perpetuate such a phenomenon by stimulating fresh N application irrespective of Net Residue. Redirecting these funds toward legacy N crediting—where recommended N rates are discounted against measured soil mineralizable N reserves—would enable farmers to draw upon this accumulated capital without a yield penalty, thereby reducing future surplus and associated Losses. For instance, the 2024 fertilizer subsidy program ($8.1 billion allocated) in Thailand could transition from blanket price supports to performance‐linked incentives. This would allow negative‐Residue districts to qualify for soil replenishment bonuses, while high‐Residue zones would earn rebates only if they demonstrably curtail application rates.

By jointly evaluating Loss and Net Residue, our framework provides a novel, spatially explicit diagnosis of N management in Asian rice systems, transcending single‐axis metrics focused only on recovery or environmental Loss. Nevertheless, estimation of fertilizer N fate is subject to some uncertainty, primarily due to limitations in current simulation frameworks (Text S5 and Figure S15). Despite such limitations, the high‐resolution gridded maps of Loss and Net Residue comprehensively provide actionable data for stakeholders to prioritize interventions in critical hotspots. Leveraging our fertilizer N fate maps, region‐tailored optimization strategies (e.g., improved fertilizer‐derived N retention in soils in Southeast Asia, optimized fertilizer formulations in East Asia) should be codified and evaluated for economic‐ecological tradeoffs. Moving forward, as agricultural datasets grow exponentially in resolution and dimensionality, this APE‐corrected continental‐scale framework can evolve from its current static baseline mapping toward a dynamic, archetype‐specific decision‐support system. By embedding the six N‐fate typologies into crop models and coupling them with spatially explicit cost–benefit functions, the framework would deliver self‐optimizing, seasonally adaptive N management prescriptions that adjust recommended rates and loss‐mitigation measures in real time as soil N capital and Loss risks shift.

## 3 Conclusion

This study reveals that Asian rice systems confront a dual threat: substantial fertilizer N losses and a pervasive imbalance in the net retention of fertilizer‐derived N in soil. By introducing Net Residue as a diagnostic metric that rigorously accounts for APE, we correct the systematic overestimations of soil N replenishment inherent in conventional budgets. Across the region, less than 7% of applied N is effectively retained as Net Residue, while nearly half (48%) is lost to the environment. This inefficiency imposes annual Nr damage costs of approximately US$98.53 billion, concentrated in India and China. Furthermore, our spatially explicit Loss–Net Residue framework classifies 42% of the rice cultivation area into distinct risk archetypes, highlighting the inadequacy of uniform management strategies. By mechanistically linking fertilizer N fate to environmental damage and management archetypes, this work delivers a scientifically grounded, region‐specific basis for decoupling rice production from fertilizer N‐driven environmental degradation and sustaining long‐term soil productivity.

## 4 Experimental Section

### 4.1 Methodological Derivation

To measure fertilizer N efficiency in crop production, the N difference and ^15^N tracer method are commonly used to quantify the percentage of fertilizer N recovered in aboveground crop biomass [19, 46]. The N difference method assesses ANRE by evaluating the difference in plant N uptake between plots treated with fertilizer N (Nuptake_(t)_, kg N ha^−1^) and control plots without N application (Nuptake_(c)_, kg N ha^−1^) (Figure 1a):

(1) $$\begin{array}{c} ANRE=\frac{Nuptake_{\left(t\right)}-Nuptake_{\left(c\right)}}{N\;rate}\times 100\% \end{array}$$

where *N rate* denotes the fertilizer N application rate in the rice growing season (kg N ha^−1^). Alternatively, the ^15^N tracer technique is used to directly measure the amount of N uptake derived from ^15^N‐labled fertilizer N (Ndff, kg N ha^−1^), thereby facilitating the calculation of ^15^N recovery efficiency (^15^NRE):
(2)

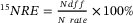




Discrepancies between ANRE and ^15^NRE have been partly attributed to the added N interaction [12, 47], commonly known as the APE. Here, we defined APE as a metric that represents the change in N uptake derived from soil N induced by fertilizer N application (ΔNdfs, kg N ha^−1^), which denotes the influence of applied N on both the availability of indigenous soil N and the ability of crop root to acquire soil N. “Apparent” denotes that the metric integrates all fertilizer‐driven pathways increasing soil‐derived N (priming, displacement, root/microbial stimulation), rather than isolating N mineralization. The APE was calculated using the following equation:

(3) $$\begin{array}{c} APE=\frac{Ndfs_{\left(t\right)}-Ndfs_{\left(c\right)}}{N\;rate}\times 100\% \end{array}$$

in which Ndfs_(t)_ and Ndfs_(c)_ are the amounts of aboveground crop N uptake (grain + straw) derived from soil for fertilized and unfertilized treatments (in kg N ha^−1^), respectively. Considering environmental N sources (e.g., biological N fixation (BNF), deposition, irrigation water) are relatively unaffected by fertilizer N inputs, we assumed equivalent contributions of the sources to both fertilized and unfertilized treatments (see Texts S1 and S5). APE is therefore calculated as follows:

(4) $$\begin{array}{c} APE=\frac{Nuptake_{\left(t\right)}-Ndff-Nuptake_{\left(c\right)}}{N\;rate}\times 100\% \end{array}$$

In the N difference method, the change in crop N uptake induced by applied fertilizer N, which is employed to assess ANRE, comprises two components: Ndff and ΔNdfs. However, ΔNdfs is not considered in the calculation of ^15^NRE [19]. Consequently, a distinct numerical relationship can be formulated between ^15^NRE and ANRE:

(5)

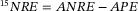




In addition to crop uptake, a portion of N fertilizer applied to rice fields either remains in the soil or is released into the environment through gaseous and hydrological processes. Therefore, the difference method is commonly utilized to assess the field‐scale proportion of fertilizer N losses (Figure 1c):

(6)

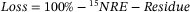

in which Residue refers to the proportion of fertilizer‐derived ^15^N retained within the top layer (0–20 cm) of the soil (kg N ha^−1^). Our previous studies have found that the residual N in the 0–20 cm layer, which is absorbed readily by subsequent‐season rice, constitutes over 70% of fertilizer N retained within the 100 cm soil profile [48]. Thus, ^15^N‐labeled fertilizer‐derived N detected in the topsoil was defined as in‐season residual N, whereas that below 20 cm was designated as fertilizer N lost to the environment. Where topsoil Residue data were missing, values from other soil depths were converted to 0–20 cm equivalents using depth‐specific conversion coefficients (Table S2). Rice root N uptake from fertilizer (Residue_(root)_, kg N ha^−1^) was included in the residual N pool to account for the stubble retention in soil after harvest. Based on the root‐to‐shoot N uptake ratio of 0.12 ± 0.07 (from 64 paired field observations), missing root fertilizer‐derived N uptake (Ndff_(root)_, kg N ha^−1^) was estimated from measured aboveground N uptake derived from fertilizer N (Ndff, kg N ha^−1^). Hence, the Residue was calculated via Equation (7).

(7) $$\begin{array}{c} Residue=\frac{Residue_{\left(soil\right)}+Residue_{\left(root\right)}}{N\;rate}\times 100\% \end{array}$$

Given that APE offsets the contribution of residual fertilizer‐derived N to replenish the soil N pool, the Net Residue was computed using the following equation:

(8) $$\begin{array}{c} \mathrm{Net}\;\mathrm{Resi}\mathrm{due}=\mathrm{Resi}\mathrm{due}-\mathrm{APE} \end{array}$$

here, Net Residue represents the net contribution of fertilizer N to the soil N pool after accounting for priming‐induced native N removal. It should be noted, however, that Net Residue does not capture the full impact of fertilization on the total soil N balance. Through dual substitution of variables, which refers to the replacement of ^15^NRE with the difference between ANRE and APE, and redefining Residue as the summation of Net Residue and APE, we derived Equation (9) from Equation (6). The reformulation depicts the complementary relationships among ANRE, Net Residue, and Loss.

(9) $$\begin{array}{c} ANRE+Net\;Residue+Loss=100\% \end{array}$$

### 4.2 Data Compilation

To clarify the divergence between ANRE and ^15^NRE and quantify fertilizer N fate in rice systems, we established a comprehensive Asian dataset from field studies through systematic literature curation (Figure S1). Peer‐reviewed articles published before June 2023 and reporting either fertilizer N recovery efficiency or fertilizer N fate in rice fields were retrieved from the Web of Science (Thomson Reuters) and the China National Knowledge Infrastructure (CNKI). The keywords used as search terms included: ‘nitrogen use efficiency’ or ‘nitrogen recovery’ or ‘fertilizer nitrogen fate’, and ‘rice’. Studies were selected if they met all the following criteria: (1) field experiments with rice cultivation concerning the fate of ^15^N labeled fertilizer or N recovery efficiency; (2) the N form input was synthetic fertilizer without organic manure incorporation; (3) at least one of the in‐season datasets on the following indices was reported: ANRE, ^15^NRE, Residue, and Loss (Figure S2). Ultimately, a comprehensive dataset was constructed by the compilation of field observational data from 109 peer‐reviewed ^15^N tracer micro‐plot studies, encompassing 97 field sites across Asian rice systems. After removing outliers (|z| > 3), we retained 378 sets of measured APE, of which 307 sets were calculated from paired ANRE and ^15^NRE via Equation 4, and 383 paired Residue and Loss observations (Figures S3 and S4). These data allowed comparison between ANRE and ^15^NRE, as well as fertilizer N fate analysis. Furthermore, to improve the comprehension of the spatial variability of ANRE across Asian rice fields, we added 5584 sets of ANRE measurements from 536 macro‐plot trials employing the N difference approach.

To quantify the spatial drivers of fertilizer N fate in rice systems, we compiled a site‐specific dataset integrating edaphic, climatic, and agronomic variables that regulate soil‐plant N dynamics. The geographic coordinates (latitude and longitude), N fertilizer application rate (N rate, kg ha^−1^), growing period (date of sowing or transplanting to date of harvest), and soil properties, including bulk density (BD, g cm^−3^), clay content (Clay, %), cation exchange capacity (CEC, cmol kg^−1^), pH measured in a soil‐water solution (−log(H^+^)), soil organic carbon (SOC, g kg^−1^), total nitrogen content (TN, g kg^−1^), and carbon‐to‐nitrogen ratio (C/N) were collected for each observation. Data were obtained from published text, tables, or digitized from figures using WebPlotDigitizer 4.5 (https://automeris.io/). The missing soil property data were extracted from the Harmonized World Soil Database Version 2.0 (HWSD v2.0, https://gaez.fao.org/pages/hwsd) via geographic coordinates. Furthermore, information on climate conditions during rice growth periods, including daily mean temperature (TMP, °C), diurnal temperature range (DTR, °C), precipitation (PRE, mm month^−1^), and potential evapotranspiration (PET, mm day^−1^), was obtained from the Climatic Research Unit gridded Time Series Version 4.07 (CRU TS v4.07) dataset (https://crudata.uea.ac.uk/cru/data/hrg/). Additionally, a long‐term and high‐resolution global gridded photosynthetically active radiation product (https://dx.doi.org/10.11888/RemoteSen.tpdc.271909) was used to extract PAR data (W m^−2^) for each site [49, 50].

### 4.3 Model Development and Performance

Environmental variables—including synthetic fertilizer N application rate (N rate), climatic conditions (TMP, DTR, PRE, PET, PAR), and soil physicochemical properties (BD, Clay, CEC, pH, SOC, TN, C/N)—were used as features to predict APE, Residue, and ANRE values using machine learning models. We assessed the performance of six machine learning algorithms encompassing: 1) linear parametric models (multiple linear regression and elastic net); 2) tree‐based ensembles (random forest [RF] and extreme gradient boosting); 3) kernel‐based regression (support vector machine); and 4) artificial neural networks (multilayer perceptrons) for predicting ANRE, APE, and Residue via five‐fold cross‐validation. Data were preprocessed with z‐score normalization after removing zero‐variance predictors and missing values, then split randomly into training (80%) and testing (20%) subsets, with the latter dedicated to measuring model generalizability. The optimal hyperparameter set was identified via Gaussian Process‐based Bayesian optimization, with the root mean square error (*RMSE*) serving as the minimization objective during 30 iterations. We developed various machine learning models using the ‘*tidymodels*’ package in R 4.4.1 (R Foundation for Statistical Computing, Vienna, Austria), with model performance assessed on the test set based on the coefficient of determination (*R^2^*) and *RMSE* (Table S3). Initially, RF was selected as the final model based on the superior predictive accuracy among six candidate approaches. Optimized RF models were trained with the following hyperparameters: 1000 decision trees and *mtry* values of 2 (ANRE and APE), 3 (Residue), 10 (Net Residue), and 5 (Loss). On the testing set, RF models exhibited moderate predictive performance, with *R^2^* values of 0.492–0.564 and *RMSE* values of 6%–11% across target variables.

RF is a machine learning algorithm based on growing an ensemble of decision trees for classification or regression [51]. To enhance the reliability of predictive outcomes and quantify associated uncertainties, the training cohorts were generated through a 2000‐iteration bootstrap resampling approach with replacement from the complete field observation dataset, followed by the establishment of an individual RF model on each of the 2000 bootstrapped samples using the best‐performing set of hyperparameters using the ‘randomForest’ package [31]. The ensemble RF models demonstrated robust predictive accuracy, with *R^2^* values ranging from 0.667 to 0.745, and *RMSE* ranging from 4% to 9%, across target variables (Figure S5). Performance was validated against field observations using ensemble mean predictions from 2000 bootstrap iterations. The representativeness of the training dataset and the robustness of the RF models were further evaluated through a series of complementary diagnostics (for full details, see Texts S6 and S7, Figures S16–S20, and Table S4). Permutation‐based feature importance was estimated by the bootstrap‐aggregated mean increase in mean square error (MSE) following feature perturbation, and its 95% confidence interval (CI) was defined as the range between  2.5th and 97.5th percentiles derived from the distribution of bootstrap replicates.

The robustness of the RF models was further corroborated by an independent, mass‐balance‐based validation. According to the N conservation principle, fertilizer‐derived N loss can be calculated as Loss = 100% − ANRE − Net Residue (Figure 1c). Using this relationship, we combined spatially explicit predictions of ANRE, APE, and Residue from the RF models to derive a gridded Loss estimate without directly training on Loss observations. The mass‐balance‐derived Loss exhibited predictive accuracy comparable to that of the RF model trained directly on measured Loss values (*R^2^* = 0.677 vs. 0.725; *RMSE* = 10% vs. 9%) (Figures S5 and S21). This convergence between the data‐driven and mass‐balance‐constrained estimates provides robust quantitative evidence that the predicted patterns of fertilizer N fate are internally consistent and mechanistically coherent. Given its marginally superior predictive accuracy, we ultimately selected the direct RF‐based Loss predictions to generate the final gridded dataset for subsequent cost–benefit analyses and N management evaluations. Nevertheless, we highlight the substantial utility of the mass‐balance approach. By unlocking vast repositories of widely available ANRE field observations, it offers a highly accessible, cost‐effective pathway for estimating fertilizer N loss at regional scales. As future studies further delineate the multi‐scale drivers and spatial heterogeneity of APE and Residue, this mass‐balance methodology holds strong promise for broad application. A detailed description of the mass‐balance framework and its broader implications for regional N loss assessment is provided in Text S8.

The relationships between key independent variables and dependent variables (APE, Residue, Net Residue, Loss, and ANRE) were analyzed through a multi‐model inference framework implemented in R. For each response variable, we systematically evaluated five candidate model structures: 1) linear regression, 2) generalized additive models, 3) exponential models, 4) logarithmic models, and 5) segmented regression with breakpoint estimation. All models were fitted using maximum likelihood estimation with the ‘mgcv’, ‘nlme’, and ‘segmented’ packages. Continuous predictors underwent rigorous outlier screening using z‐score normalization [52]. Observations with absolute z‐scores > 3.0 were identified as statistical outliers and excluded from subsequent analyses. The optimal model for each dependent variable was selected through an information‐theoretic approach based on the Akaike Information Criterion (*AIC*) and the Bayesian Information Criterion (*BIC*). Final models were evaluated further using predictive accuracy metrics: mean absolute error (*MAE*) and *RMSE* for goodness‐of‐fit, and *R^2^* (McFadden's pseudo‐*R^2^* for non‐linear models) to quantify variance explained.

### 4.4 Gridded Prediction

To estimate fertilizer N fate across Asian rice systems using RF regression models, we constructed a high‐resolution prediction dataset by integrating the distribution of rice cultivation areas across Asia and gathering gridded cell‐specific environmental predictor data (Figure S6). First, we identified rice cultivation grids using the Spatial Production Allocation Model dataset (SPAM2020; 5 arc‐min resolution; https://doi.org/10.7910/DVN/SWPENT), retaining those with non‐zero harvested area, and subsequently converted to points denoting locations for predictions [53]. The synthetic fertilizer N application rates for rice in 2020, derived from the global crop‐specific N fertilization dataset (https://doi.org/10.11888/Terre.tpdc.300446) [54, 55], were determined as the N rate for the prediction locations. The mean values of the gridded data of synthetic fertilizer N application rates were used as the N rate for each respective level of administrative region, including county, district, state, province, and country. Afterward, missing N rate values were imputed using the mean values in ascending order of administrative rank. For countries without data, the N rate was supplemented by the crop N application rates provided by FAO STAT. Consequently, the N rates for Bhutan, Laos, Brunei, Kazakhstan, and Timor‐Leste were established at 8.54, 24.02, 24.30, 2.51, and 0.37 kg ha^−1^ year^−1^, respectively. Subsequently, geographic distribution information on prediction locations was used to extract the peak planting months during the main rice‐growing season from the database of rice calendars (RiceAtlas, https://dx.doi.org/10.7910/DVN/JE6R2R) [56]. The meteorological data sourced from CRU TS v4.07 (https://crudata.uea.ac.uk/cru/data/hrg/cru_ts_4.07/), which spans the years 2011 to 2020, along with the PAR product covering the period from 2011 to 2017, were averaged to provide a comprehensive representation of climatic conditions over the decade. We assumed that rice had a growing period of four months. Consequently, climatic variables corresponding to the rice growing period for each prediction location were obtained by calculating the average over the four months commencing with the peak planting month. For double‐cropping rice regions, climate variables were calculated as the mean of both growing seasons. Based on the attributed database in HWSD v2.0 (https://www.fao.org/soils‐portal/data‐hub/soil‐maps‐and‐databases/harmonized‐world‐soil‐database‐v20/en/), the weighted mean of soil parameters in the 0–20 cm layer was calculated using the share in soil mapping unit as weights. Raster image of soil mapping units at 30 arc‐second resolution was resampled using a majority algorithm to gridded data with 5 arc‐min resolution. Furthermore, we matched the rice prediction sites with the values of soil mapping units based on the geographic location, and then linked them to the attribute database to acquire soil physicochemical properties for each gridded cell.

The environmental covariates were incorporated into the bootstrap‐aggregated ensemble RF framework comprising 2000 iterations as feature variables to predict APE, Residue, Net Residue, Loss, and ANRE in Asian rice systems. Following this, the 95% CI was constructed using the percentile method, where the lower and upper bounds for each predictor variable at every prediction target were derived from the 2.5th and 97.5th percentiles of the distribution of outputs generated through 2000 bootstrap iterations. Ultimately, the point‐based predicted outputs with corresponding 95% CI underwent geostatistical transformation into raster maps with a resolution of 5 arc minutes using ArcGIS Pro (ESRI, Redlands, California, USA) (Figure S15). Employing the APE‐mediated methodological framework, we have generated the first high‐resolution map decoding the spatial distribution of fertilizer‐derived Net Residue and Loss in Asian rice systems.

### 4.5 Cost‐Benefit Analysis

In Asian rice cultivation systems with intensive synthetic N fertilizer inputs, economic sustainability is challenged not only by direct input costs of N fertilizer but also by environmental costs stemming from reactive N (Nr) emissions. We calculated the country‐specific net agroeconomic benefits (Net benefit, $ yr^−1^) by deducting the direct input costs of N fertilizer (Fertilizer cost, $ yr^−1^) and the environmental costs of Nr losses (Nr cost, $ yr^−1^) from the rice yield benefit (Yield benefit, $ yr^−1^):

(10) $$\begin{array}{c} Net\;benefit=Yield\;benefit-Fertilizer\;cost-Nr\;cost \end{array}$$

The yield benefit was computed for each country using the following equation:

(11) $$\begin{array}{c} Yield\;benefit=Yield\times Area_{harvest}\times Price_{grain} \end{array}$$

in which country‐specific rice grain yield (Yield, kg ha^−1^), harvested area (Area_harvest_, ha) and producer price (Price_grain_, $ kg^−1^) were extracted from FAOSTAT (Data S1) [9].

At 5‐arcmin resolution, we calculated synthetic fertilizer N inputs (N_fer_, kg N yr^−1^) and losses (N_loss_, kg N yr^−1^):

(12) $$\begin{array}{c} N_{fer,g}=N_{rate,g}\times Area_{harvest,g} \end{array}$$

(13) $$\begin{array}{c} N_{loss,g}=N_{fer,g}\times Loss_{g} \end{array}$$

where Area_harvest,g_ denotes the rice harvested area in grid *g*, derived from the SPAM2020 dataset. Subsequently, country‐level totals of N_fer_ and N_loss_ were determined through the Summary Statistics tool in ArcGIS Pro.

Fertilizer price data were derived from the UN Comtrade Database (https://comtrade.un.org/) by dividing urea export values (FOB, USD) by the corresponding net export weights (kg), and then converted to fertilizer N price (Price_fer_, $ kg^−1^ N) based on urea's N content (46%). Subsequently, the cost of N fertilizer inputs in country *i* was calculated according to the following equation:

(14) $$\begin{array}{c} Fertilizer\;cost=N_{fer,i}\times Price_{fer,i} \end{array}$$

Furthermore, the environmental costs of Nr losses (Nr cost, $ yr^−1^) were estimated based on the predicted Loss, share of Nr in total losses [57, 58], and the damage cost per unit of Nr [6]. We estimated economic cost to the whole society resulting from synthetic fertilizer‐derived Nr losses from rice cultivation, considering damages to climate (CCost_i,j_), health (HCost_i,j_), and ecosystem services (ECost_i,j_) [6], as shown in the following equations:

(15) $$\begin{array}{c} Nr\;cost_{i}=CCost_{i}+HCost_{i}+ECost_{i} \end{array}$$

(16) $$\begin{array}{c} CCost_{i}=\sum_{j}N_{loss,i}\times Share_{Nr,j}\times Price_{Nr,ccost,i,j} \end{array}$$

(17) $$\begin{array}{c} HCost_{i}=\sum_{j}N_{loss,i}\times Share_{Nr,j}\times Price_{Nr,hcost,i,j} \end{array}$$

(18) $$\begin{array}{c} ECost_{i}=\sum_{j}N_{loss,i}\times Share_{Nr,j}\times Price_{Nr,ecost,i,j} \end{array}$$

where *j* enumerates Nr forms in the emission pathway, including gaseous emissions (NH_3_, N_2_O, NO_X_) and hydrologic losses (runoff, leaching). All prices were converted to constant 2020 U.S. dollars using the Consumer Price Index (CPI) for inflation adjustment. The national‐specific data on the Nr partitioning coefficient and the damage cost per unit of Nr emission can be found in Table S5 and Data S1 in the Excel file, respectively.

### 4.6 Assessing N Management Status along Net Residue‐Loss Dimensions

The complementary relationships among ANRE, Net Residue, and Loss indicate that sustainable N management requires concurrent control of fertilizer N retention and reduction of fertilizer N losses. To assess N management status in Asian rice systems, we characterized fertilizer N fate along two dimensions: Net Residue and Loss magnitudes. Net Residue reflects the net effect of fertilizer N on soil N pools: negative Net Residue (indicating soil N depletion) occurs when APE surpasses Residue; near‐equilibrium states arise when APE is approximately equal to Residue; excessive Net Residue reduces in‐season N use efficiency and creates residual N pools vulnerable to subsequent loss. The upper quartile criterion is widely used to delineate agronomic “hot spots” [59, 60]. We therefore classified rice systems based on Net Residue thresholds: negative (<0 kg N ha^−^^1^ yr^−^^1^), low (0–16 kg N ha^−^^1^ yr^−^^1^), and high (>75th percentile: 16 kg N ha^−^^1^ yr^−^^1^) (Figure S13). Similarly, Loss was categorized as low (≤75th percentile: 96 kg N ha^−^^1^ yr^−^^1^) or high (>96 kg N ha^−^^1^ yr^−^^1^). Ultimately, the cross‐classification yields six rice system types: LLNR, LLLR, LLHR, HLNR, HLLR, and HLHR. Targeting the efficient LLLR rice system as the benchmark, we developed tailored optimization strategies for other rice system types based on their distinct N fate profiles.

## Author Contributions

X.Z. conceived the study, designed the overall research strategy, and supervised the project. X.L. assembled and harmonized the meta‐dataset. X.L. and S.C. developed the analysis pipeline and code. X.L. and S.C. carried out the statistical analyses and generated the figures and schematics. X.Y. contributed to methodology design and advised on protocols and model specification. L.X. and J.L. contributed to external validation and sensitivity analyses and to manuscript revisions. X.L. and S.C. wrote the original draft. X.L., S.C., X.Z., L.X., J.L., and X.Y. reviewed and edited the manuscript. All authors approved the final version and agree to be accountable for all aspects of the work.

## Funding

This work was supported by the National Natural Science Foundation of China (32402694, 42277331), the Natural Science Foundation of Jiangsu Province (BK20241700), the Frontier Project from the Institute of Soil Science, Chinese Academy of Sciences (ISSASIP2406), the Young Elite Scientists Sponsorship Program by CAST (2023QNRC001), the High‐level Talent Training Project of Jiangsu Province (20243‐0920), and Henan Xinlianxin Chemicals Group Co., LTD.

## Conflicts of Interest

The authors declare no conflicts of interest.

## Supporting information




**Supporting File 1**: advs76227‐sup‐0001‐SuppMat.docx.

**Supporting File 2**: advs76227‐sup‐0002‐Data.xlsx.

## Data Availability

The data that support the findings of this study are available in the supplementary information of this article.
