# Supplementary material for: Correcting Apparent Priming Bias Unveils Fertilizer Nitrogen‐Risk Archetypes of Surplus and Depletion Across Asian Rice Systems
Source: Adv Sci (Weinh). 2026 Jun 22;13(48):e76227. doi: 10.1002/advs.76227 (PMC13337020; doi:10.1002/advs.76227)
Supplement: Supplementary file 1 [file ADVS-13-e76227-s002.docx]

Supporting Information

Correcting Apparent Priming Bias Unveils Fertilizer Nitrogen-Risk Archetypes of Surplus and Depletion across Asian Rice Systems

Xiuyun Liu,^#^ Siyuan Cai,^#^ Longlong Xia, Jagdish K. Ladha, Xiaoyuan Yan, Xu Zhao*

E-mail: zhaoxu@issas.ac.cn

**This file includes:**

Supporting Text 1 to 8

Figures S1 to S21

Tables S1 to S5

SI References

**Other supporting materials for this manuscript include the following:**

Dataset S1

# Supporting Text

## Text S1. Discrepancies between ANRE and ^15^NRE

Our results confirmed that apparent nitrogen recovery efficiency (ANRE) consistently exceeded ^15^N recovery efficiency (^15^NRE), which was partly attributed to the positive apparent priming effect (APE) (Figure 1b). Therefore, from an agronomic perspective, it is more preferable to adopt ANRE for evaluating current-season effects of synthetic N fertilizer,^[1]^ as it accurately reflects the actual degree of improvement in plant N nutrition induced by fertilizer N addition, including the increase in N uptake derived from soil.

In contrast, ^15^NRE isolated fertilizer-derived uptake alone, systematically underestimating the agronomic value of synthetic N by disregarding APE-driven soil N accessibility. This oversight has critical implications. Overreliance on ^15^NRE risks misguiding fertilizer recommendations, as it fails to capture the full extent of N utilization efficiency, potentially encouraging excessive application to compensate for perceived inefficiencies. The results underscore the necessity of revising efficiency frameworks to incorporate plant-soil synergies, particularly in regions prioritizing sustainable intensification.

The addition of ^15^N-labeled N sources may displace indigenous unlabeled N from soil pools, such as mineral-fixed ammonium and microbial biomass,^[2-4]^ and it may also serve as a substitute for indigenous unlabeled N in processes such as immobilization, nitrification, and denitrification.^[5]^ These coupled effects collectively drive the accelerated release of soil indigenous inorganic N.^[6]^ Additionally, the concurrent presence of mineral N derived from synthetic fertilizer and labile C from root exudates significantly accelerates soil organic matter decomposition, mainly by activating r-strategist microorganisms, which consequently boosts the production of extracellular enzymes.^[7]^ Furthermore, N fertilizer exerts profound effects on rice root systems,^[8]^ which in turn amplify plant capacity to exploit indigenous soil N pools through both morphological adaptations and rhizosphere biochemical interactions.^[5]^ BNF in rice soils is often suppressed under synthetic-N inputs and enhanced in unfertilized plots.^[9]^ Therefore, unequal BNF between treatments can bias interpretation: as BNF_ctrl_ > BNF_fert_, part of the discrepancies between ANRE and ^15^NRE reflects BNF suppression.,

Notably, the observed divergence reflects both APE and methodological scale/design differences, and should be interpreted accordingly. ANRE is usually estimated from large field plots, whereas ^15^NRE often relies on microplots. Differences in plot size, borders, tracer dilution, and disturbance can bias either metric up or down and contribute to ANRE-^15^NRE gaps.^[10]^ Moreover, ANRE is a difference-from-control in the same season and can be confounded by long-term changes in soil fertility (residual N, build-up/depletion) and carryover effects, whereas ^15^NRE is less sensitive to baseline shifts but inherits tracer-specific biases. Thirdly, ^15^NRE distinguishes sources (soil vs fertilizer) but may miss unmeasured pools (like BNF); ANRE aggregates all sources but cannot apportion them.

**Text S2. Spatial pattern and environmental drivers of ANRE**

This is the first time that a continental-scale ANRE surface for rice has been resolved using machine-learning algorithms. ANRE ranged from 26 to 61% of applied N (Figure S8b, Supporting Information). Beyond the overriding control exerted by N application rate, the Random Forest (RF) model assigned substantial importance to soil total N (TN), cation exchange capacity (CEC), and pH, together explaining about a half of the variance (Figure S8a, Supporting Information). Efficiencies cluster where these edaphic filters converge: high CEC, modest TN, and a near-neutral pH favor ANRE values above 45%. Such conditions prevail in northeast China, Japan, central India, and the Iranian and Anatolian basins (Figure S6, Supporting Information). Mechanistically, abundant exchange sites stabilize NH_4_^+^ against Loss, and a buffered pH minimizes volatilization while maintaining rhizosphere enzyme activity.^[11]^ Together, these traits allow moderate N dressings to be used with near-optimal efficiency.

**Text S3. Cost-benefit analysis of synthetic fertilizer N input**

Reactive nitrogen (Nr) emissions to the environment impose a range of adverse impacts on ecosystem services.^[12]^ For nitrogen oxides (NOx), these include damage to buildings from acid deposition, ozone-induced injury to crops and forests, and loss of plant biodiversity from N enrichment. For ammonia (NH_3_), ecosystem damage comprises damage to buildings from particulates and loss of plant biodiversity. For nitrous oxide (N_2_O), enhanced ultraviolet radiation resulting from stratospheric ozone depletion adversely affects crop production. For N runoff to surface freshwaters, ecosystem damage encompasses declining waterfront property values, loss of recreational use, loss of endangered species, and increased eutrophication.

Unit ecosystem damage costs of reactive nitrogen (Nr) emissions were obtained from Sobota et al. (2015), whose low, median, and high estimates were directly adopted to define three valuation scenarios: a low-cost scenario, a baseline (median-cost) scenario, and a high-cost scenario.^[12]^ These estimates were first converted to constant 2020 US dollars using the US Consumer Price Index and then adjusted to individual countries following the willingness-to-pay (WTP)-based approach of Gu et al.^[13]^ The adjustment is performed as:

$$\begin{aligned} {Price}_{Nr,ecost,i,j}={Price}_{Nr,ecost,US,j}\times\frac{{WTP}_{i}}{{WTP}_{US}} \end{aligned}(1)$$

where *j* represents the form of Nr emission, including gaseous emissions (NH_3_, N_2_O, NO_x_) and hydrologic losses (runoff, leaching); Price_Nr,ecost,US,_*_j_* is the unit ecosystem damage cost of Nr emission in the United States expressed in the constant 2020 U.S. dollars; WTP*_i_* and WTP_US_ are the willingness-to-pay values for ecosysytem service in country *i* and the United States, respectively.

Nr emissions from agricultural systems also impose substantial damage on both human health and the climate system. NH_3_ is a major precursor of fine particulate matter (PM₂.₅), impairing respiratory and cardiovascular function and contributing to premature mortality.^[14]^ NO_x_ similarly promotes PM₂.₅ formation and tropospheric ozone production, further exacerbating adverse health outcomes. N losses to water bodies via runoff and leaching contaminate drinking water supplies, posing risks of nitrate-induced health effects.^[12]^ The country-specific unit health damage costs of NH_3_ and NO_x_ emissions from synthetic fertilizer N were quantified through atmospheric chemistry transport modeling coupled with economic parameters,^[15]^ these estimates are considered to be of high reliability.

With respect to climate, N_2_O is a potent long-lived greenhouse gas with a 100-year global warming potential approximately 273 times that of carbon dioxide,^[16]^ while NH_3_ and NO_x_ also exert indirect climate effects through aerosol formation and atmospheric chemistry. Notably, NH_3_-derived aerosols can induce net cooling, meaning that reducing NH₃ emissions may partially offset the climate benefits achieved through N_2_O mitigation.^[13]^ Country-specific unit damage cost coefficients for the climate impacts of NH_3_, N_2_O, and NO_x_ were sourced from Gao et al. (2025) and used as the baseline scenario values.^[15]^ The corresponding underestimation and overestimation scenarios were derived by scaling the baseline values according to the low-to-median and high-to-median ratios reported in Sobota et al. (2015), respectively.^[12]^ This unified three-scenario framework (low, baseline, and high) enables a consistent assessment of the sensitivity of the total societal cost of Nr emissions to uncertainty in damage cost valuations across both ecosystem and climate impact categories.

Country-level fluxes of individual synthetic fertilizer-derived Nr loss pathways were calculated by applying the loss factors in Table S5 (Supporting Information) to national-scale Loss estimates. Under the baseline scenario, the total environmental cost attributed to Nr losses amounted to US$98.53 billion yr⁻¹ (Figure 3d). Notably, the climate impact reflects a net cooling effect: NH₃ volatilization and NO_X_ emissions from paddies generate sulfate-neutralizing aerosols that scatter incoming solar radiation, partially offsetting warming from N_2_O and other greenhouse gases and yielding a net cooling benefit of US$4.05 billion yr⁻¹. This benefit is, however, outweighed substantially by fertilizer cost (US$9.82 billion yr⁻¹), ecosystem degradation costs (US$13.79 billion yr⁻¹) and human health costs (US$88.79 billion yr⁻¹), resulting in a net societal benefit of US$261.98 billion yr⁻¹ (Figure S11d, Supporting Information). Under the low-cost scenario, total environmental costs declined to US$83.25 billion yr⁻¹ (ecosystem damage: US$5.02 billion yr⁻¹; climate benefit: US$10.56 billion yr⁻¹), while under the high-cost scenario, total environmental costs increased to US$108.27 billion yr⁻¹ (ecosystem damage: US$22.57 billion yr⁻¹; climate benefit: US$3.08 billion yr⁻¹) (Figure S12, Supporting Information).

**Text S4. Fertilizer N input drives N use efficiency across Asia**

ANRE declined sharply once synthetic N inputs exceeded rice demand. The LLNR, LLLR, and LLHR clusters exhibited a high ANRE (47% ± 3%, 46% ± 4%, and 46% ± 4%, respectively) with modest N inputs (<200 kg N ha^−1^ yr^−1^). Country-level patterns mirrored this relationship. The Philippines, China’s double-rice belt, and Vietnam’s triple-rice Mekong delta routinely exceeded 250 kg N ha^−1^ seasonally yet achieved less than 40% ANRE in the HLHR pattern (Figure S14, Supporting Information). By contrast, Indonesia, Cambodia, and Myanmar, with low N input dominated by single‑season (<150 kg N ha^−1^ yr^−1^), post the region’s best ANRE (>45%). Notably, regions with N input surpassing 300 kg N ha^−1^ yr^−1^ still manifested negative Net Residue, underscoring that increasing N application alone cannot arrest soil N mining. The persistence of negative residues under high inputs reflected the offsetting effect of the APE: as fertilizer N rose, microbially driven mineralization of native organic N was stimulated, thereby masking the gross fertilizer contribution to the soil N pool.

**Text S5. Uncertainty analysis**

The upper and lower bounds of the 95% confidence interval (CI) for fertilizer N losses demonstrated geospatial coherence across Asian rice systems (Figure S15, Supporting Information). However, we identified spatial disparities in prediction uncertainty, manifesting as the narrower width of 95% CI in China compared to in the Southeast Asian counterparts. The RF models for APE, Residue, and ANRE incorporated training datasets with 52% (N = 198), 71% (N = 272), and 84% (N = 4922) of observations originating from Chinese rice systems, respectively (Figure S3, Supporting Information). This imbalanced representation of training samples in RF models resulted in superior predictive accuracy for fertilizer N fluxes in Asia.

The estimation of fertilizer N losses in Asian rice systems is subject to some uncertainty, primarily due to limitations in current simulation frameworks. Key variables such as rice cultivar and site-specific fertilization practices (e.g., timing, placement, irrigation interactions) remain unrepresented in models, largely owing to sparse empirical data. For instance, rice cultivars exhibit substantial genetic variability in N use efficiency, with ANRE ranging from 10% to 90% under conventional management.^[17]^ While cultivar-mediated differences in N use efficiency and root architecture still contribute to variability in N loss pathways, rice cultivar selection had no statistically significant influence on ANRE compared to climatic and edaphic factors.^[17]^ Similarly, site-specific agronomic practices—such as deep placement of urea (which reduces volatilization losses by 11–65% compared to broadcast application) or water management interactions—are excluded due to insufficient empirical data on implementation rates and environmental outcomes.^[18]^ Irrigation regime is a first-order control on residue-loss partitioning. Continuous flooding sustains reducing conditions that slow SOM mineralization and can increase net residue but promote NH_3_ volatilization; alternate wetting and drying enhances nitrification-denitrification coupling, often reducing NH_3_ yet raising N_2_O and leaching risks, and can lower Net Residue by accelerating soil-N turnover.^[19]^ Prioritizing field experiments that quantify genotype × environment × management interactions could substantially reduce such uncertainties and improve predictive accuracy.

Notably, we report fertilizer-attributed Net Residue as the difference between the fertilizer-derived N residue rate (Residue) and APE. By design, Net Residue excludes exogenous N inputs—BNF, atmospheric deposition, irrigation-borne and organic N—so as not to confound fertilizer-induced processes with background or management-responsive N sources. Our conclusions pertain to fertilizer-attributed dynamics rather than the full N budget. Including plausible BNF/deposition credits may shift a subset of negative Net Residue grids toward neutrality/positivity, while cells with large negative Net Residue would remain risk-flagged unless exogenous inputs are substantial. We highlight these shifts to distinguish where improved fertilizer stewardship alone is sufficient versus where additional N credits (e.g., BNF enhancement) are required to avoid soil N drawdown.

Regarding the broader impact of fertilizer N on the native soil N pool, we acknowledge that while incorporating the APE effectively corrects the overestimation of fertilizer N retention (Residue) in soil, it does not perfectly encapsulate the absolute net N balance. This is because fertilizer application accelerates the mineralization of native soil organic N, and a portion of this “primed” native mineral N is inevitably lost to the environment (i.e., primed soil N loss) rather than being entirely absorbed by plants. Therefore, our “Net Residue” metric (Residue minus APE) does not perfectly equate to the absolute real-world replenishment of the soil N pool. However, previous study demonstrated that under fertilized conditions, soil background losses accounted for only 31% of the total Nr losses in Chinese rice paddies.^[20]^ Furthermore, the long-term 15N tracing experiments in a highly intensified rice–wheat double-cropping system indicated that residual N immobilized in the soil acts as a slow-release N source, exhibiting a significantly lower environmental loss rate compared to freshly applied fertilizer N.^[21]^

## Text S6. Evaluation of sample representativeness

To elucidate N dynamics in rice-soil system, this study integrated observational data from ^15^N tracer micro-plot experiments—covering fertilizer‑derived N fate (^15^NRE, Residue, and Loss)—with field-measured ANRE. APE was derived by pairwise subtraction of ANRE from ¹⁵NRE. To assess the representativeness of the compiled database for capturing N dynamics across Asian rice systems, we systematically evaluated three key indicators—APE (n = 378), Residue (n = 383, paired with Loss), and ANRE (n = 5,891)—with respect to their temporal coverage, spatial distribution, and environmental covariate space.

### Temporal representativeness

Regarding the experimental timeline, the proportion of experiments conducted after the year 2000 for the APE, Residue, and ANRE metrics reached 65%, 78%, and 92%, respectively (Figure S4, Supporting Information). The past three decades have coincided with the rapid advancement of modern agriculture, the widespread adoption of chemical fertilizers, and the maturation of ^15^N isotope tracing techniques. Consequently, although the experimental data in our database span over four decades, they remain highly representative of contemporary agricultural conditions. Furthermore, this 40-year temporal span is strategically advantageous for capturing the impacts of diverse interannual climatic variations on paddy N dynamics.

### Spatial representativeness

Based on Tobler’s First Law of Geography, environmental factors such as climatic conditions, soil properties, and field management practices exhibit inherent spatial autocorrelation.^[22]^ Climatological and pedological studies have explicitly demonstrated that macro-scale soil moisture—fundamentally driven by regional atmospheric forcings such as precipitation and evaporation patterns—exhibits a robust spatial autocorrelation scale of approximately 500 km.^[23]^ Furthermore, adopting buffers of 100 km or more around representative sites to delineate “Technology Extrapolating Domains” is a standard methodological paradigm in global agricultural mapping, particularly for topographically homogeneous crop-producing plains.^[24]^ Considering that Asian rice production is geographically highly agglomerated in specific river basins and coastal deltas—areas characterized by consistent topography, climates, soil properties, and irrigation regimes—we utilized geographical buffers (radii of 50, 100, 150, 200, and 250 km) around the sampling sites to define their spatial footprints (Table S4, Supporting Information). By comparing these footprints against the actual spatial intensity of Asian rice cultivation (harvested area and total production), we quantitatively assessed the geographic representativeness of the observations.

The spatial coverage analysis reveals that a 200-km buffer for the ANRE dataset captures up to 67% of the total harvested area and 72% of the total rice production in Asia (Table S4, Supporting Information). For the APE and Residue datasets derived directly from ^15^N tracer experiments, the coverage for harvested area and production within a 200-km radius ranges from 33% to 41%. Given the high financial and logistical costs associated with continuous ^15^N field-tracing, globally available observational data remain intrinsically limited. To our knowledge, the database constructed herein encompasses the largest and most comprehensive collection of ^15^N tracer observations for Asian rice paddies currently available^[25]^. Considering the high methodological stability of the ^15^N technique and this substantial agricultural footprint (capturing over one-third of the continent’s production capacity), the included APE and Residue data are deemed adequately representative for regional-scale evaluations.

### Environmental covariate space representativeness

This study employed a RF algorithm to upscale localized field observations. For the training dataset, site-specific soil properties and N application rates were extracted directly from previous literature. Any missing soil and growing-season climatic variables were gap-filled using the Harmonized World Soil Database Version 2.0 (HWSD v2.0) and the Climatic Research Unit (CRU TS v4.07) dataset, based on precise geographic coordinates. For the prediction grids (pan-Asian dataset), variables were extracted directly from spatial datasets. To mitigate potential biases introduced by extreme weather anomalies, climatic variables for the prediction grids were calculated as a 10-year average (2011–2020) over the rice growing season.^[26]^

Importantly, probability density distributions revealed a high degree of congruence in the environmental covariate space between the training and prediction datasets. We quantified this alignment using the Kolmogorov–Smirnov (K-S) test. The K-S test, like traditional null hypothesis significance testing, is profoundly sensitive to sample size: with training samples ranging from 378 to 5891 and prediction grids exceeding one hundred thousand, even statistically negligible distributional discrepancies will inherently yield highly significant *p*-values (*p* < 0.001).^[27]^

Consequently, to avoid the “large sample *p*-value problem,” we rely fundamentally on the K-S *D* statistic—the maximum vertical distance between cumulative distribution functions, which serves as a robust effect size—to assess substantive overlap in the covariate space.^[28]^ For critical soil parameters driving the RF models, the *D* statistics were modest: 0.12–0.26 for cation exchange capacity (CEC), 0.10–0.22 for clay content, and 0.09–0.21 for pH. For key climatic variables such as photosynthetically active radiation (PAR) and mean temperature (TMP), *D* values ranged from 0.19 to 0.34. These relatively low D values indicate that the training and prediction distributions are largely coincident across the dominant environmental gradients of Asian rice fields.

The *D* statistic for N application rate (N rate) was marginally higher (0.34–0.42), reflecting the fact that experimental sites are disproportionately concentrated in regions with elevated N inputs (e.g., China and India), with training data spanning 20–500 kg N ha⁻¹. Notably, the prediction domain includes a non-negligible fraction of grid cells with N rates below 20 kg N ha⁻¹, a range underrepresented in the training data. Nevertheless, this underrepresentation is unlikely to substantially bias regional-scale extrapolations for two reasons. First, agronomic evidence demonstrates that at N inputs below 20 kg ha⁻¹, the magnitude and variability of fertilizer N fate and soil N dynamics remain intrinsically limited. Second, the *D* values for N rate, while higher than those for other covariates, remain within a range that does not indicate severe distributional divergence. Moreover, regions where extrapolation uncertainty is elevated due to low N-rate coverage are explicitly delineated in the accompanying uncertainty maps, ensuring transparent communication of confidence limits.

Collectively, the low K-S *D* statistics across the suite of environmental predictors confirm that the training dataset adequately spans the core feature space of the prediction domain, thereby supporting the geographical transferability of the trained RF models.^[29]^

## Text S7. Rationale and robustness assessment of the Random Forest framework

Agroecological datasets are frequently characterized by high-dimensional, nonlinear, and interactive relationships among covariates. Under such conditions—particularly when sample sizes are limited—conventional parametric models often fail to adequately resolve underlying distributional patterns and their environmental determinants owing to stringent assumptions regarding degrees of freedom and residual structure.^[30]^ RF, by contrast, is a nonparametric ensemble learning algorithm that circumvents these constraints. The algorithm operates by training individual decision trees on bootstrap samples drawn with replacement from the original data. At each node split, only a randomly selected subset of predictors is considered, which decorrelates the constituent trees. Through this deliberate injection of randomness and subsequent ensemble averaging, RF achieves enhanced generalization capacity and stability without requiring the sample size to vastly exceed the number of predictors.^[31]^

To maximize the predictive robustness and credibility of N dynamic indicators in the rice–soil system—metrics that are jointly modulated by soil properties, climatic conditions, and field management practices—we implemented a rigorous machine learning pipeline specifically designed to accommodate modest sample sizes. The pipeline comprised the following sequential steps. First, the dataset was partitioned via hold-out validation into a training set (80%) and an independent test set (20%). Within the training set, a five-fold cross-validation framework was established to guide hyperparameter tuning. Bayesian optimization was then employed to identify optimal hyperparameter configurations for six candidate regression algorithms: Multiple Linear Regression, Elastic Net, RF, Extreme Gradient Boosting, Support Vector Machine, and Multilayer Perceptron. Algorithm performance was subsequently evaluated on the held-out test set, with selection favoring the model that maximized the coefficient of determination (R²) and minimized root mean square error (*RMSE*). RF consistently emerged as the optimal algorithm across four N dynamic indicators examined—ANRE, Residue, Net Residue, and Loss—achieving test-set R² values ranging from 0.502 to 0.564 and *RMSE* values from 6% to 11% (Table S3, Supporting Information). The final RF model was trained on the full training dataset using the optimized hyperparameter configuration and was subsequently employed for spatial extrapolation to 0.5-arcminute grids covering Asian rice fields. To quantify pixel-level uncertainty, a 2,000-iteration Bootstrap ensemble was executed, yielding both the ensemble mean prediction and the associated 95% confidence interval.

Several complementary analyses were conducted to evaluate the fidelity, generalizability, and ecological coherence of the RF model. First, predictions for the training samples yielded R² values ranging from 0.667 to 0.745 and *RMSE* values from 4% to 9%, indicating that the model successfully captured the dominant systematic signals within the meta-dataset and exhibited no evidence of underfitting (Figure S5, Supporting Information). Second, probability density distributions of key soil and climatic predictors exhibited substantial overlap between the training locations and the full prediction domain, supporting the geographical transferability of the trained model (Figures S16–S18, Supporting Information). We note that prediction uncertainty is elevated in regions where fertilizer nitrogen application rates fall below the minimum observed in the training data (<20 kg ha⁻¹); these zones of reduced confidence are explicitly demarcated in the accompanying uncertainty maps, thereby ensuring transparent communication of extrapolation limits (Figure S15 Supporting Information). Third, a feature sensitivity analysis based on out-of-bag (OOB) cross-validation revealed that OOB-R² increased rapidly with the inclusion of the first several most important predictors and subsequently plateaued as additional covariates were incorporated (Figure S19, Supporting Information). This plateau provides empirical evidence that the model does not conflate stochastic noise with ecological signal, thereby ruling out overfitting.

Finally, partial dependence analysis demonstrated that the RF model successfully recovered mechanistically plausible relationships between paddy N dynamics and key environmental covariates (Figure S20, Supporting Information). For instance, increasing CEC and clay content were associated with reduced APE, consistent with enhanced ammonium adsorption by soil minerals and greater physical protection of soil organic N, which collectively attenuate fluctuations in mineral N supply following fertilizer addition. As N application rate increased, both ANRE and Residue declined while gaseous and hydrological losses escalated, reflecting the saturation of crop N demand and soil immobilization capacity, with excess N being routed to environmental loss pathways. These coherent, theoretically interpretable marginal response curves confirm that the RF algorithm is capturing genuine agroecological signals rather than fitting spurious noise, thereby substantiating the reliability of the high-resolution spatial extrapolations presented in this study.

To further assess the credibility of our RF-based spatial predictions, we cross-validated the modeled fertilizer N fate indicators and risk archetypes against independent, long-term field experiments and ^15^N tracer monitoring data from two representative fertilizer-derived N Loss hotspots: the Changshu site in eastern China and the Pantnagar site in northern India.^[21,32]^ The former, a long-term ^15^N micro-plot experiment located in Jiangsu province and representative of a highly intensified rice–wheat double-cropping system, has demonstrated that under conventional high-N inputs (e.g., 300 kg N ha⁻¹ per season), approximately half of the applied fertilizer N is lost to the environment during the first rice season, with multi-decadal observations further confirming that in-season losses account for over 91% of the total cumulative fertilizer N loss over a 17-year period. Our RF model predicted a mean in-season fertilizer N loss rate of 53% (± 3%) for Jiangsu Province in 2020 (Figure 3a), and the spatial diagnostic framework classified the majority of the provincial paddy fields into HLHR or HLLR (High-Loss, High/Low-Net Residue) archetypes—consistent with the documented reality of massive N inputs driving substantial environmental N leakage (Figure 4a). The Pantnagar site, located in the Indo-Gangetic Plain, hosts a representative long-term rice–wheat rotation system. Decadal agronomic evaluations at this site have revealed a persistent downward trend in crop yields, attributed largely to the continuous depletion of the native soil N pool resulting from imbalanced urea broadcasting and inefficient nutrient management. Our spatially explicit framework specifically diagnosed the Pantnagar grid cells as HLLR or HLNR (High-Loss, Low/Negative-Net Residue) archetypes (Figure 4a), signifying that external N inputs are insufficient to offset the accelerated mineralization and subsequent plant uptake of native soil N. This classification closely mirrors the empirically observed trajectory of soil N depletion and declining yield potential documented at this site.

Collectively, these independent, first-order validations across two highly representative Asian agroecosystems strongly confirm that our integrated modeling framework accurately captures both regional-scale N fluxes and the underlying biogeochemical dynamics, thereby substantially reinforcing the credibility of the high-resolution spatial predictions presented in this study.

**Text S8. Decode fertilizer nitrogen loss across Asian rice systems: leveraging apparent priming effect**

Accounting for the various pathways of losses, N fertilizer-induced losses are conventionally quantified by subtracting rice N uptake and soil residues from the N application amount using ^15^N tracer field trials, rather than by direct measurement at the field scale. However, it is not feasible to estimate the losses of fertilizer N at regional scale through extensive field trials, due to the time-consuming and resource-intensive nature of the ^15^N tracer methods. Regional-scale N loss assessments commonly integrate complementary approaches, including empirical emission factor methods (e.g., IPCC Tier 1) and process-based biogeochemical models (e.g., DeNitrification-DeCompositon (DNDC) model), yet inevitably encounter trade-offs between oversimplification of spatial heterogeneity and excessive mechanistic complexity.^[33,34]^ There is an urgent need for a convenient and robust method for estimating fertilizer N losses across Asian rice production.

Inferring fertilizer-derived N loss from the mass-balance relationship Loss = 100% − ANRE − Net Residue is both feasible and mechanistically justified, because it is grounded in the conservation of N within a closed soil–crop budget (Figure 2c). In principle, the fraction of applied fertilizer N that is neither recovered by the crop nor retained in the soil must be lost to the environment through gaseous emissions, leaching, runoff, or other pathways. To implement this framework at the regional scale, we constructed RF models for ANRE—leveraging extensive field-trial observations from the N-difference method—as well as for APE and Residue, both of which are constrained by measurements from ¹⁵N microplot trials. These models were then used to generate spatially explicit predictions of ANRE, APE, and Residue across Asian rice systems. Net Residue was subsequently derived as the difference between the gridded Residue and APE estimates. Finally, a gridded map of fertilizer-derived N loss across Asian rice systems was obtained by subtracting ANRE and Net Residue from 100% according to the mass-balance equation. The comparable predictive performance of this mass-balance-derived Loss estimate (*R²* = 0.677; *RMSE* = 10%) relative to a model trained directly on observed Loss values (*R²* = 0.725; *RMSE* = 9%) further supports the robustness and credibility of this approach (Figures S5 and S21, Supporting Information).

By using APE as a bridging variable, this framework harnesses the extensive historical datasets of ANRE from widely available N-difference field trials for fertilizer N loss assessment, thereby effectively circumventing the prohibitive cost and chronic data scarcity associated with direct ^15^N-labeled loss measurements at the regional scale. Moreover, by decomposing the total loss into mechanistically distinct drivers—crop recovery, soil retention, and the priming effect—the approach yields predictions that are internally consistent with the soil–crop N budget. This transparency enhances interpretability and safeguards against implausible extrapolations that purely data-driven models may produce. Future studies that elucidate the mechanisms underlying the soil N priming effect and the characteristics of fertilizer N retention in soils, and that clarify the quantitative relationship between ANRE and Loss, hold the potential to transform this mass-balance method into a convenient, robust, and mechanistically grounded shortcut for quantifying fertilizer N losses at the regional scale.

**Figures**

**Figure S1.** Schematic of the workflow.

**Figure S2.** PRISMA flow diagram showing the selection process for studies.

**Figure S3.** Locations of field trial sites in meta-analysis. (a) Apparent priming effect (APE). (b) Proportion of residual synthetic fertilizer-derived nitrogen (Residue). (c) Apparent nitrogen recovery efficiency (ANRE).

**Figure S4.** Temporal distribution and cumulative frequency of the field experiment for key nitrogen dynamics variables. (a) Apparent priming effect (APE). (b) Proportion of residual synthetic fertilizer-derived nitrogen (Residue). (c) Apparent nitrogen recovery efficiency (ANRE). The histograms (left y-axis) illustrate the number of observations conducted in specific experiment years, while the red lines with markers (right y-axis) represent the cumulative percentage of the dataset over time.

**Figure S5.** Performance of Random Forest regression models. APE, Apparent priming effect; Residue, Proportion of residual synthetic fertilizer-derived nitrogen; ANRE, Apparent nitrogen recovery efficiency; Net Residue, Proportion of net residual synthetic fertilizer-derived nitrogen; Loss, Proportion of fertilizer nitrogen loss. *RMSE* and *R²* denote the root-mean-square error and coefficient of determination, respectively, calculated from paired observed and predicted values. N represents number of observations.

**Figure S6.** Spatial distribution of environmental factors. Fertilizer factors: N rate, nitrogen application rate (kg ha^−1^); Climatic factors: TMP, daily mean temperature (℃); DTR, diurnal temperature range (℃); PAR, photosynthetically active radiation (w m^−2^); PRE, precipitation (mm month^−1^); PET, potential evapotranspiration (mm day^−1^); Edaphic factors: BD, bulk density (g cm^−3^); Clay, percent by weight clay (%); CEC, cation exchange capacity (cmol kg^−1^); pH, pH measured in a soil-water solution (−log(H^+^)); SOC, soil organic carbon (g kg^−1^); TN, total nitrogen content (g kg^−1^); C/N, carbon-to-nitrogen ratio. Synthetic N rates were taken from the 2020 global crop-specific fertilization inventory. Decadal (2011–2020) climate data were derived from CRU‑TS v4.07, with PAR averaged over the period from 2011 to 2017. Surface (0–20 cm) soil properties were derived from HWSD v2.0 (see Methods).

**Figure S7.** Effects of environmental and management factors on the apparent priming effect (APE) and fertilizer nitrogen residue (Residue) resolved by a multi-model regression framework. The solid lines are regression lines (*p* < 0.05). Black dashed lines denote non-significant regressions (*p* ≥ 0.05). The grey area around the solid line shows the 95% confidence intervals of the estimations. Fertilizer factors: N rate, nitrogen application rate (kg N ha^−1^); Climatic factors: TMP, daily mean temperature (℃); DTR, diurnal temperature range (℃); PAR, photosynthetically active radiation (w m^−2^); PRE, precipitation (mm month^−1^); PET, potential evapotranspiration (mm day^−1^); Edaphic factors: BD, bulk density (g cm^−3^); Clay, percent by weight clay (%); CEC, cation exchange capacity (cmol kg^−1^); pH, pH measured in a soil-water solution (−log(H^+^)); SOC, soil organic carbon (g kg^−1^); TN, total nitrogen content (g kg^−1^); C/N, carbon-to-nitrogen ratio.

**Figure S8.** Spatial distribution of apparent nitrogen recovery efficiency (ANRE) driven by environmental factors. (a) Relative importance of environmental and management factors driving variation in ANRE identified by Random Forest regression. (b) Gridded ANRE predicted by Random Forest regression. (c) Effects of factors on ANRE resolved by multi-model regression. The solid lines are regression lines (*p* ≥ 0.05). Black dashed lines denote non-significant regressions (*p* < 0.05). The grey area around the solid line shows the 95% confidence intervals of the estimations. Fertilizer factors: N rate, nitrogen application rate (kg N ha^−1^); Climatic factors: TMP, daily mean temperature (℃); DTR, diurnal temperature range (℃); PAR, photosynthetically active radiation (w m^−2^); PRE, precipitation (mm month^−1^); PET, potential evapotranspiration (mm day^−1^); Edaphic factors: BD, bulk density(g cm^−3^); Clay, percent by weight clay (%); CEC, cation exchange capacity (cmol kg^−1^); pH, pH measured in a soil-water solution (−log(H^+^)); SOC, soil organic carbon (g kg^−1^); TN, total nitrogen content (g kg^−1^); C/N, carbon-to-nitrogen ratio.

**Figure S9.** Impacts of environmental factors on synthetic fertilizer-derived nitrogen net residue (Net Residue). (a) Relative importance of environmental and management factors driving variation in Net Residue identified by Random Forest regression. (b) Effects of environmental and management factors on Net Residue resolved by a multi-model regression framework. The solid lines are regression lines (*p* < 0.05). Black dashed lines denote non-significant regressions (*p* ≥ 0.05). The grey area around the solid line shows the 95% confidence intervals of the estimations. Fertilizer factors: N rate, nitrogen application rate (kg N ha^−1^); Climatic factors: TMP, daily mean temperature (℃); DTR, diurnal temperature range (℃); PAR, photosynthetically active radiation (w m^−2^); PRE, precipitation (mm month^−1^); PET, potential evapotranspiration (mm day^−1^); Edaphic factors: BD, bulk density(g cm^−3^); Clay, percent by weight clay (%); CEC, cation exchange capacity (cmol kg^−1^); pH, pH measured in a soil-water solution (−log(H^+^)); SOC, soil organic carbon (g kg^−1^); TN, total nitrogen content (g kg^−1^); C/N, carbon-to-nitrogen ratio.

**Figure S10.** Impacts of environmental factors on synthetic fertilizer-derived loss (Loss). (a) Relative importance of environmental and management factors driving variation in Loss identified based on Random Forest regression. (b) Effects of environmental and management factors on Loss resolved by a multi-model regression framework. The solid lines are regression lines (p < 0.05). Black dashed lines denote non-significant regressions (p ≥ 0.05). The grey area around the solid line shows the 95% confidence intervals of the estimations. Fertilizer factors: N rate, nitrogen application rate (kg N ha^−1^); Climatic factors: TMP, daily mean temperature (℃); DTR, diurnal temperature range (℃); PAR, photosynthetically active radiation (w m^−2^); PRE, precipitation (mm month^−1^); PET, potential evapotranspiration (mm day^−1^); Edaphic factors: BD, bulk density (g cm^−3^); Clay, percent by weight clay (%); CEC, cation exchange capacity (cmol kg^−1^); pH, pH measured in a soil-water solution (−log(H^+^)); SOC, soil organic carbon (g kg^−1^); TN, total nitrogen content (g kg^−1^); C/N, carbon-to-nitrogen ratio.

**Figure S11.** Fate of fertilizer nitrogen (N) and associated benefits and costs in Asian rice production at the country level. (a) Spatial distribution of synthetic fertilizer-derived N loss ratio. (b) Pathways for fertilizer-derived N losses. Percentages above the light blue and light grey bars show the proportion of reactive N losses and total N losses to the input, respectively. (c) Spatial distribution of net benefits rate associated with synthetic N fertilizer. (d) Results of cost-benefit analysis of N fertilizer. Percentages above the blue dots show the proportions of net benefit to yield benefit. The horizontal labels of plot (d) correspond with plot (b). The top-ten rice-producing Asian countries ranked by descending 2020 output: China (CN), India (IN), Bangladesh (BD), Indonesia (ID), Vietnam (VN), Thailand (TH), Myanmar (MM), Philippines (PH), Pakistan (PK), and Cambodia (KH) (FAO 2022).

**Figure S12.** Cost–benefit analysis of synthetic fertilizer nitrogen input to Asian rice production under low- and high-cost valuation scenarios. Rice production benefit and reactive nitrogen (Nr) emission costs for the top ten rice-producing Asian countries under the low-cost scenario (a) and high-cost scenario (b). The inset donut charts display each country's share of total fertilizer-derived Nr emissions damage costs from Asian rice systems under the respective valuation scenario. Breakdown of costs and net benefit under the low-cost scenario (c) and high-cost scenario (d), including fertilizer cost, ecosystem damage cost, human health damage cost, climate impact, and net societal benefit. In all panels, positive values denote economic gains (rice production benefit or net benefit), while negative values denote costs. Climate impact appears as a positive value in the breakdown, reflecting a net cooling effect from ammonia- and nitrogen oxide-derived aerosols that partially offsets warming from nitrous oxide. All monetary values are expressed in constant 2020 US dollars. The top-ten rice-producing Asian countries ranked by descending 2020 output: China (CN), India (IN), Bangladesh (BD), Indonesia (ID), Vietnam (VN), Thailand (TH), Myanmar (MM), Philippines (PH), Pakistan (PK), and Cambodia (KH) (FAO 2022).

**Figure S13.** Classification threshold for Net Residue and Loss level. Shown are kernel density distributions of Net Residue and Loss. Net Residue values below zero are defined as negative net residue (NR). Using the 75th percentile as a threshold, the data are categorized into low/high residuals (LR/HR) and low/high losses (LL/HL). The upper-quartile criterion isolates the most extreme 25% of observations and is widely used to delineate agronomic “hotspots” of nutrient surplus or risk.^[35,36]^

**Figure S14.** Fertilizer nitrogen (N) inputs and use efficiency in the top-ten rice-producing countries of Asia. Box plots show the distribution of apparent N recovery efficiency (ANRE), with the black solid points indicating nation-specific mean ANRE. Bar graphs depict nation-specific mean annual fertilizer N application rates, and error bars denote standard errors. LLNR, Low-Loss-negative-Net Residue; LLLR, Low-Loss-low-Net Residue; LLHR, Low-Loss-high-Net Residue; HLNR, High-Loss-negative-Net Residue; HLLR, High-Loss-low-Net Residue; HLHR, High-Loss-high-Net Residue. The top-ten rice-producing Asian countries ranked by descending 2020 output: China (CN), India (IN), Bangladesh (BD), Indonesia (ID), Vietnam (VN), Thailand (TH), Myanmar (MM), Philippines (PH), Pakistan (PK), and Cambodia (KH) (FAO 2022).

**Figure S15.** Distribution of prediction uncertainty across Asian rice fields. APE, Apparent priming effect; Residue, Proportion of residual synthetic fertilizer-derived nitrogen; ANRE, Apparent nitrogen recovery efficiency; Net Residue, Proportion of residual synthetic fertilizer-derived nitrogen; Loss, Proportion of fertilizer nitrogen loss. The difference between the upper and lower limits of the 95% confidence interval is defined as the width of the 95% confidence interval.

**Figure S16.** Environmental feature space representativeness of the training dataset compared to the regional prediction domain for apparent priming effect (APE). Probability density functions (PDFs) of 13 key environmental covariates are compared between the model training samples (blue area, n=378) and the entire Asian rice cultivation grids (pink area, prediction samples, n=103,385). The Kolmogorov-Smirnov (K-S) test statistic (D) and p-values are inset in each panel. While the p-values are inherently sensitive and statistically significant (p<0.001) due to the overwhelmingly large sample size of the background prediction grids, the relatively low D statistics and the high visual congruence of the density curves confirm exceptional representativeness. The training sites successfully encompass the full multidimensional climatic, pedological, and management gradients of the Asian rice systems, confirming that the Random Forest models are interpolating within a well-represented feature space rather than blindly extrapolating.

**Figure S17.** Environmental feature space representativeness of the training dataset compared to the regional prediction domain for Residue. Probability density functions (PDFs) of 13 key environmental covariates are compared between the model training samples (blue area, n=383) and the entire Asian rice cultivation grids (pink area, prediction samples, n=103,385). The Kolmogorov-Smirnov (K-S) test statistic (D) and p-values are inset in each panel. While the p-values are inherently sensitive and statistically significant (p<0.001) due to the overwhelmingly large sample size of the background prediction grids, the relatively low D statistics and the high visual congruence of the density curves confirm exceptional representativeness. The training sites successfully encompass the full multidimensional climatic, pedological, and management gradients of the Asian rice systems, confirming that the Random Forest models are interpolating within a well-represented feature space rather than blindly extrapolating.

**Figure S18.** Environmental feature space representativeness of the training dataset compared to the regional prediction domain for apparent nitrogen recovery efficiency (ANRE). Probability density functions (PDFs) of 13 key environmental covariates are compared between the model training samples (blue area, n=5891) and the entire Asian rice cultivation grids (pink area, prediction samples, n=103,385). The Kolmogorov-Smirnov (K-S) test statistic (D) and p-values are inset in each panel. While the p-values are inherently sensitive and statistically significant (p<0.001) due to the overwhelmingly large sample size of the background prediction grids, the relatively low D statistics and the high visual congruence of the density curves confirm exceptional representativeness. The training sites successfully encompass the full multidimensional climatic, pedological, and management gradients of the Asian rice systems, confirming that the Random Forest models are interpolating within a well-represented feature space rather than blindly extrapolating.

**Figure S19.** Feature elimination sensitivity analysis demonstrating the robustness and anti-overfitting capacity of the tuned Random Forest models. APE, Apparent priming effect; Residue, Proportion of residual synthetic fertilizer-derived nitrogen; ANRE, Apparent nitrogen recovery efficiency; Net Residue, Proportion of net residual synthetic fertilizer-derived nitrogen; Loss, Proportion of fertilizer nitrogen loss. The plots show the Out-of-Bag (OOB) *R^2^* trajectories for the key nitrogen dynamics metrics as the number of included environmental covariates increases (ranked by importance). The vertical dashed lines indicate the optimal number of features where performance peaks.

**Figure S20.** Bootstrapped partial dependence (PDP) plots illustrating the isolated marginal effects of the top five driving environmental covariates on regional nitrogen dynamic indicators. APE, Apparent priming effect; Residue, Proportion of residual synthetic fertilizer-derived nitrogen; ANRE, Apparent nitrogen recovery efficiency; Net Residue, Proportion of net residual synthetic fertilizer-derived nitrogen; Loss, Proportion of fertilizer nitrogen loss. Solid colored lines represent the mean marginal predictions across 200 independent Bootstrap iterations. The surrounding gray shaded areas delineate the 95% confidence intervals.

**Figure S21.** Predictive performance of the mass-balance-derived Net Residue and Loss estimates. Net Residue, defined as the proportion of net residual synthetic fertilizer-derived nitrogen (N), was calculated as the difference between Residue and APE. Loss, representing the proportion of fertilizer-derived N loss, was derived by subtracting ANRE and Net Residue from 100% according to the mass-balance equation (Loss = 100% − ANRE − Net Residue). *RMSE* and *R²* denote the root-mean-square error and coefficient of determination, respectively, calculated from paired observed and predicted values. N represents number of observations.

**Tables**

**Table S1.** Nomenclature and definitions of key variables and model parameters.

| **Category** | **Abbreviation** | **Full name** | **Definition** | **Unit** |
| --- | --- | --- | --- | --- |
| Nitrogen fate variables | ANRE | Apparent nitrogen recovery efficiency | Proportion of the incremental aboveground crop nitrogen uptake attributed to fertilizer application | % |
|  | ^15^NRE | ^15^N recovery efficiency | Proportion of applied ¹⁵N-labeled fertilizer recovered in aboveground crop biomass | % |
|  | APE | Apparent priming effect | Additional soil-derived nitrogen taken up by the crop in response to fertilizer nitrogen application | % |
|  | Residue | Fertilizer-derived nitrogen residue in soil | Proportion of applied ¹⁵N-labeled fertilizer remaining in the soil at harvest | % |
|  | Net Residue | Net residual nitrogen | Net retention of fertilizer-derived nitrogen in soil after accounting for APE; calculated as Residue − APE | % |
|  | Loss | Fertilizer-derived nitrogen loss | Proportion of applied fertilizer nitrogen lost to the environment | % |
| Fertilizer nitrogen-risk archetypes | HLHR | High-Loss-High-Net Residue | Intensive systems with high environmental N losses and high net retention of fertilizer-derived nitrogen in soil | - |
|  | HLLR | High-Loss-Low-Net Residue | Systems with high environmental nitrogen losses and low net retention of fertilizer-derived nitrogen in soil | - |
|  | HLNR | High-Loss-Negative-Net Residue | Systems with high environmental nitrogen losses and a net deficit of fertilizer-derived nitrogen in soil | - |
|  | LLHR | Low-Loss-High-Net Residue | Systems with low environmental nitrogen losses and high net retention of fertilizer-derived nitrogen in soil | - |
|  | LLLR | Low-Loss-Low-Net Residue | Efficient systems with low environmental nitrogen losses and low net retention of fertilizer-derived nitrogen in soil | - |
|  | LLNR | Low-Loss-Negative-Net Residue | Systems with low environmental nitrogen losses but a net deficit of fertilizer-derived nitrogen in soil | - |
| Model performance metrics | R^2^ | Coefficient of determination | Proportion of variance in observed values explained by model predictions | - |
|  | RMSE | Root-mean-square error | Square root of the mean squared differences between predicted and observed values; reported in the same unit as the predicted variable | % |

**Note:** All nitrogen fate variables are expressed as percentages of the total synthetic fertilizer nitrogen applied (kg N ha^−1^).

**Table S2.** Coefficients to standardize fertilizer N residual proportion to 0–20 cm.

| **0–15 cm** | **0–25 cm** | **0–30 cm** | **0–40 cm** | **0–50 cm** | **0–60 cm** | **0–80 cm** |
| --- | --- | --- | --- | --- | --- | --- |
| 1.10 | 0.95 | 0.90 | 0.80 | 0.76 | 0.71 | 0.70 |

Depth-specific scaling coefficients were derived from our database containing field-observed ^15^N tracer measurements across the Asian rice system.

**Table S3.** Comparisons of the model performance across six machine learning algorithms

| **Methods** | **APE^a)^ (%)** | | **Residue (%)** | | **Net Residue (%)** | | **Loss (%)** | | **ANRE (%)** | |
| --- | --- | --- | --- | --- | --- | --- | --- | --- | --- | --- |
|  | **R^2 b)^** | **RMSE** | **R^2^** | **RMSE** | **R^2^** | **RMSE** | **R^2^** | **RMSE** | **R^2^** | **RMSE** |
| Multiple Linear Regression | 0.233 | 12 | 0.119 | 8 | 0.293 | 14 | 0.288 | 14 | 0.063 | 13 |
| Elastic Net | 0.234 | 12 | 0.121 | 8 | 0.307 | 14 | 0.323 | 13 | 0.063 | 13 |
| Random Forest | 0.492 | 9 | **0.502** | **6** | **0.533** | **11** | **0.564** | **10** | **0.555** | **9** |
| Extreme Gradient Boosting | 0.448 | 10 | 0.361 | 7 | 0.492 | 12 | 0.517 | 11 | 0.541 | 9 |
| Support Vector Machine | **0.511** | **9** | 0.394 | 6 | 0.522 | 12 | 0.523 | 11 | 0.506 | 10 |
| Multilayer Perceptrons | 0.397 | 10 | 0.433 | 6 | 0.497 | 12 | 0.529 | 11 | 0.123 | 13 |

**^a)^** APE, apparent priming effect; Residue, proportion of residual fertilizer nitrogen; Net Residue, proportion of net residual fertilizer nitrogen; Loss, proportion of fertilizer nitrogen losses; ANRE, apparent nitrogen recovery efficiency;

**^b)^** *R^2^*, regression coefficients of determination; *RMSE*, root mean square error.

**Table S4.** Spatial representativeness analysis of the observation sites across varying buffer radii for key nitrogen dynamics variables.

| **Variable** | **Buffer radius (km)** | **Harvested area coverage^b)^ (%)** | **Production coverage (%)** |
| --- | --- | --- | --- |
| APE**^a)^** | 50 | 5 | 6 |
|  | 100 | 15 | 17 |
|  | 150 | 24 | 27 |
|  | 200 | 33 | 36 |
|  | 250 | 40 | 43 |
| Residue | 50 | 7 | 9 |
|  | 100 | 18 | 22 |
|  | 150 | 28 | 33 |
|  | 200 | 36 | 41 |
|  | 250 | 42 | 47 |
| ANRE | 50 | 22 | 27 |
|  | 100 | 41 | 47 |
|  | 150 | 55 | 61 |
|  | 200 | 67 | 72 |
|  | 250 | 75 | 78 |

**^a)^** APE, apparent priming effect; Residue, proportion of residual fertilizer nitrogen; ANRE, apparent nitrogen recovery efficiency

**^b)^** The background distribution maps for Asian rice harvested area and production were derived from the Spatial Production Allocation Model dataset (SPAM2020; 5 arc-min resolution; [https://doi.org/10.7910/DVN/SWPENT](https://doi.org/doi:10.7910/DVN/SWPENT)). Coverage percentages represent the proportion of the total Asian rice domain captured within the respective buffers.

**Table S5.** Pathways of synthetic fertilizer N losses in rice production

| **N loss pathway** | **Loss factor^a)^ (%)** | **Partitioning coefficient^b)^ (%)** | **Source** |
| --- | --- | --- | --- |
| NH_3_ | 17.4 | 47.03 | Shang, et al.^[20]^ |
| N_2_O | 0.5 | 1.35 |  |
| NO | 0.2 | 0.54 |  |
| N leaching | 3.1 | 8.38 |  |
| N runoff | 2.3 | 6.22 |  |
| N_2_ | 13.5 | 36.48 | Xia, et al.^[37]^ |

**^a)^** Loss factor refers to the proportion of the N losses to the total synthetic fertilizer N inputs;

**^b)^** Pathway-specific portioning coefficient was defined as the fractional contribution of individual N loss to total fertilizer-induced N loss.

# Dataset S1 (separate file).

This dataset was uploaded separately in an Excel file.

**SI References**

[1]	K. Harmsen, J. T. Moraghan, A comparison of the isotope recovery and difference methods for determining nitrogen fertilizer efficiency. *Plant Soil* **1988**, *105*, 55.

[2]	D. S. Jenkinson, R. H. Fox, J. H. Rayner, Interactions between fertilizer nitrogen and soil nitrogen-the so-called 'priming' effect. *J. Soil Sci.* **1985**, *36*, 425.

[3]	G. A. Gouveia, G. D. Eudoxie, Distribution of fertiliser n among fixed ammonium fractions as affected by moisture and fertiliser source and rate. *Biol. Fertil. Soils* **2007**, *44*, 9.

[4]	R. Nieder, D. K. Benbi, H. W. Scherer, Fixation and defixation of ammonium in soils: a review. *Biol. Fertil. Soils* **2011**, *47*, 1.

[5]	F. Azam, Added nitrogen interaction in the soil-plant system-a review. *J. Agron.* **2002**, *1*, 54.

[6]	R. Gentile, B. Vanlauwe, P. Chivenge, J. Six, Interactive effects from combining fertilizer and organic residue inputs on nitrogen transformations. *Soil Biol. Biochem.* **2008**, *40*, 2375.

[7]	R. Chen, M. Senbayram, S. Blagodatsky, O. Myachina, K. Dittert, X. Lin, E. Blagodatskaya, Y. Kuzyakov, Soil c and n availability determine the priming effect: microbial n mining and stoichiometric decomposition theories. *Glob. Change Biol.* **2014**, *20*, 2356.

[8]	M. Xiao, H. Zang, T. Ge, A. Chen, Z. Zhu, P. Zhou, C. T. Atere, J. Wu, Y. Su, Y. Kuzyakov, Effect of nitrogen fertilizer on rice photosynthate allocation and carbon input in paddy soil. *Eur. J. Soil Sci.* **2019**, *70*, 786.

[9]	H. Dai, S. Wei, J. Li, W. Kong, B. Wang, J. Pei, J. Wu, Fertilization effects on symbiotic and free-living biological nitrogen fixations: similar effects but different mechanisms. *Appl. Soil Ecol.* **2024**, *202*, 105590.

[10]	T. Krupnik, J. Six, J. Ladha, M. Paine, C. Kessel, V. Kessel, "An assessment of fertilizer nitrogen recovery efficiency by grain crops across scales" in *Agriculture and the nitrogen cycle: assessing the impacts of fertilizer use on food production and the environment* (Eds: A. Mosier, J. K. Syers, J. R. Freney), Island Press **2004**, pp. 193-207.

[11]	X. J. Wang, C. X. Tang, The role of rhizosphere ph in regulating the rhizosphere priming effect and implications for the availability of soil-derived nitrogen to plants. *Ann. Bot.* **2018**, *121*, 143.

[12]	D. J. Sobota, J. E. Compton, M. L. Mccrackin, S. Singh, Cost of reactive nitrogen release from human activities to the environment in the United States. *Environ. Res. Lett.* **2015**, *10*, 25006.

[13]	B. Gu, X. Zhang, S. K. Lam, Y. Yu, H. J. M. van Grinsven, S. Zhang, X. Wang, B. L. Bodirsky, S. Wang, J. Duan, C. Ren, L. Bouwman, W. de Vries, J. Xu, M. A. Sutton, D. Chen, Cost-effective mitigation of nitrogen pollution from global croplands. *Nature* **2023**, *614*, E19.

[14]	B. Gu, L. Zhang, R. Van Dingenen, M. Vieno, H. J. Van Grinsven, X. Zhang, S. Zhang, Y. Chen, S. Wang, C. Ren, S. Rao, M. Holland, W. Winiwarter, D. Chen, J. Xu, M. A. Sutton, Abating ammonia is more cost-effective than nitrogen oxides for mitigating PM_2.5_ air pollution. *Science* **2021**, *374*, 758.

[15]	Y. Gao, J. Cui, X. Zhang, G. Hoogenboom, D. Wallach, Y. Huang, S. Reis, T. Lin, B. Gu, Cost-effective adaptations increase rice production while reducing pollution under climate change. *Nat. Food* **2025**.

[16]	Intergovernmental Panel on Climate Change (IPCC). "The Earth’s Energy Budget, Climate Feedbacks and Climate Sensitivity" In *Climate Change 2021 – The Physical Science Basis: Working Group I Contribution to the Sixth Assessment Report of the Intergovernmental Panel on Climate Change*. Cambridge University Press, (Eds: V. Masson-Delmotte, P. Zhai, A. Pirani, S. L. Connors, C. Péan, S. Berger, N. Caud, Y. Chen, L. Goldfarb, M. I. Gomis, M. Huang, K. Leitzell, E. Lonnoy, J. B. R. Matthews, T. K. Maycock, T. Waterfield, O. Yelekçi, R. Yu, B. Zhou), Cambridge University Press, Cambridge, United Kingdom and New York, NY, USA, **2021**, pp. 923-1054.

[17]	S. Cai, X. Zhao, X. Yan, Effects of climate and soil properties on regional differences in nitrogen use efficiency and reactive nitrogen losses in rice. *Environ. Res. Lett.* **2022**, *17*, 54039.

[18]	L. Zhu, H. Sun, L. Liu, K. Zhang, Y. Zhang, A. Li, Z. Bai, G. Wang, X. Liu, H. Dong, C. Li, Optimizing crop yields while minimizing environmental impact through deep placement of nitrogen fertilizer. *J. Integr. Agric.* **2025**, *24*, 36.

[19]	J. K. Ladha, C. K. Reddy, A. T. Padre, C. van Kessel, Role of nitrogen fertilization in sustaining organic matter in cultivated soils. *J. Environ. Qual.* **2011**, *40*, 1756.

[20]	Y. Shang, Y. Yin, H. Ying, X. Tian, Z. Cui, Updated loss factors and high-resolution spatial variations for reactive nitrogen losses from chinese rice paddies. *J. Environ. Manage.* **2024**, *358*, 120752.

[21]	X. Zhao, Y. Wang, S. Cai, J. K. Ladha, M. J. Castellano, L. Xia, Y. Xie, Z. Xiong, B. Gu, G. Xing, X. Yan, Legacy nitrogen fertilizer in a rice-wheat cropping system flows to crops more than the environment. *Sci. Bull.* **2024**, *69*, 1212.

[22]	W. R. Tobler, A computer movie simulating urban growth in the detroit region. *Econ. Geogr.* **1970**, *46*, 234.

[23]	J. K. Entin, A. Robock, K. Y. Vinnikov, S. E. Hollinger, S. Liu, A. Namkhai, Temporal and spatial scales of observed soil moisture variations in the extratropics. *J. Geophys. Res. Atmos.* **2000**, *105*, 11865.

[24]	J. van Wart, K. C. Kersebaum, S. Peng, M. Milner, K. G. Cassman, Estimating crop yield potential at regional to national scales. *Field Crops Res.* **2013**, *143*, 34.

[25]	Z. Quan, X. Zhang, Y. Fang, E. A. Davidson, Different quantification approaches for nitrogen use efficiency lead to divergent estimates with varying advantages. *Nat. Food* **2021**, *2*, 241.

[26]	P. Grassini, L. G. J. van Bussel, J. Van Wart, J. Wolf, L. Claessens, H. Yang, H. Boogaard, H. de Groot, M. K. van Ittersum, K. G. Cassman, How good is good enough? Data requirements for reliable crop yield simulations and yield-gap analysis. *Field Crops Res.* **2015**, *177*, 49.

[27]	M. Lin, H. C. Lucas, G. Shmueli, Research commentary—too big to fail: large samples and the p-value problem. *Inf. Syst. Res.* **2013**, *24*, 906.

[28]	G. M. Sullivan, R. Feinn, Using effect size—or why the p value is not enough. *J. Grad. Med. Educ.* **2012**, *4*, 279.

[29]	H. Meyer, E. Pebesma, Machine learning-based global maps of ecological variables and the challenge of assessing them. *Nat. Commun.* **2022**, *13*, 2208.

[30]	D. R. Cutler, T. C. J. Edwards, K. H. Beard, A. Cutler, K. T. Hess, J. Gibson, J. J. Lawler, Random forests for classification in ecology. *Ecology* **2007**, *88*, 2783.

[31]	L. Breiman, Random forests. *Mach. Learn.* **2001**, *45*, 5.

[32]	J. K. Ladha, D. Dawe, H. Pathak, A. T. Padre, R. L. Yadav, B. Singh, Y. Singh, Y. Singh, P. Singh, A. L. Kundu, R. Sakal, N. Ram, A. P. Regmi, S. K. Gami, A. L. Bhandari, R. Amin, C. R. Yadav, E. M. Bhattarai, S. Das, H. P. Aggarwal, R. K. Gupta, P. R. Hobbs, How extensive are yield declines in long-term rice–wheat experiments in asia? *Field Crops Res.* **2003**, *81*, 159.

[33]	G. Aliyu, A. Sanz-Cobena, C. Müller, M. Zaman, J. Luo, D. Liu, J. Yuan, Z. Chen, Y. Niu, A. Arowolo, W. Ding, A meta-analysis of soil background n2o emissions from croplands in china shows variation among climatic zones. *Agric. Ecosyst. Environ.* **2018**, *267*, 63.

[34]	Y. Wang, Y. Liu, L. Xia, H. Akiyama, X. Chen, J. Chen, Y. Fang, T. Vancov, Y. Li, Y. Yao, D. Wu, B. Yu, S. X. Chang, Y. Cai, Accounting for differences between crops and regions reduces estimates of nitrate leaching from nitrogen-fertilized soils. *Commun. Earth Environ.* **2025**, *6*, 29.

[35]	B. Hoppe, D. White, A. Harding, G. Mueller-Warrant, B. Hope, E. Main, High resolution modeling of agricultural nitrogen to identify private wells susceptible to nitrate contamination. *J. Water Health* **2014**, *12*, 702.

[36]	R. Kumar, F. Heße, P. S. C. Rao, A. Musolff, J. W. Jawitz, F. Sarrazin, L. Samaniego, J. H. Fleckenstein, O. Rakovec, S. Thober, S. Attinger, Strong hydroclimatic controls on vulnerability to subsurface nitrate contamination across europe. *Nat. Commun.* **2020**, *11*, 6302.

[37]	L. Xia, X. Li, Q. Ma, S. K. Lam, B. Wolf, R. Kiese, K. Butterbach Bahl, D. Chen, Z. Li, X. Yan, Simultaneous quantification of n_2_ , NH_3_ and n_2_o emissions from a flooded paddy field under different n fertilization regimes. *Glob. Change Biol.* **2020**, *26*, 2292.
